# BcDKM: Blockchain-Based Dynamic Key Management Scheme for Crowd Sensing in Vehicular Sensor Networks

**DOI:** 10.3390/s25185699

**Published:** 2025-09-12

**Authors:** Mingrui Zhang, Ru Meng, Lei Zhang

**Affiliations:** 1 Shanghai Key Laboratory of Trustworthy Computing, Software Engineering Institute, East China Normal University, Shanghai 200062, China; zhangmingrui@stu.ecnu.edu.cn; 2 Engineering Research Center of Software/Hardware Co-Design Technology and Application, Ministry of Education, East China Normal University, Shanghai 200062, China; 3 College of Cyber Security, Jinan University, Guangzhou 510632, China; mengru93@stu2021.jnu.edu.cn

**Keywords:** vehicular sensor network, crowd sensing, vehicular cloud, group key management, group key agreement

## Abstract

Vehicular sensor networks (VSNs) consist of vehicles equipped with various sensing devices, such as LiDAR. In a VSN, vehicles and/or roadside units (RSUs) can be organized into a vehicular cloud (VC) to enable the sharing of sensing and computational resources among participants, thereby supporting crowd-sensing applications. However, the highly dynamic nature of vehicular mobility poses significant challenges in terms of establishing secure and scalable group communication within the VC. To address these challenges, we first introduce a lightweight extension of the continuous group key agreement (CGKA) scheme by incorporating an administrator mechanism. The resulting scheme, referred to as CGKAwAM, supports the designation of multiple administrators within a single group for flexible member management. Building upon CGKAwAM, we propose a blockchain-based dynamic key management scheme, termed BcDKM. This scheme supports asynchronous join and leave operations while achieving communication round optimality. Furthermore, RSUs are leveraged as blockchain nodes to enable decentralized VC discovery and management, ensuring scalability without relying on a centralized server. We formally analyze the security of both CGKAwAM and BcDKM. The results demonstrate that the proposed scheme satisfies several critical security properties, including known-key security, forward secrecy, post-compromise security, and vehicle privacy. Experimental evaluations further confirm that BcDKM is practical and achieves a well-balanced tradeoff between security and performance.

## 1. Introduction

Vehicular sensor networks (VSNs) can leverage vehicles equipped with advanced onboard (sensing) devices (e.g., LiDAR, around view monitors and AI chips) to collect high-precision environmental and traffic data, thereby enabling large-scale urban crowd sensing applications (e.g., real-time traffic analysis and road anomaly detection) [1]. However, many vehicles remain stationary for extended periods (e.g., charging or parking), leaving these sensing and computing resources underutilized.

To address this issue, researchers have proposed the concept of vehicular cloud (VC) [2], deployed in the VSN environment. A VC enables a group of nearby authenticated vehicles or roadside units (RSUs) to dynamically form a cooperative sensing system, maximizing the utilization of idle sensing resources from stationary vehicles [3]. Within a VC, vehicles can act as cloud administrators or cloud members. Once the VC is established, cloud users (i.e., entities authorized to access the VC) can submit sensing tasks to the cloud administrator via end-to-end secure channels established through key agreement [4]. Upon receiving the tasks, the cloud administrator distributes them to cloud members for collaborative execution.

### 1.1. Related Work

In the early stages of VC research, the focus was primarily on the design of VC architectures and their potential applications, such as cooperative sensing and resource sharing. However, relatively limited attention was given to the secure establishment of VCs. In the following, we review the evolution of VC architectures and discuss efforts aimed at ensuring the secure establishment of VCs.

**VC Architecture.** The concept of a VC was first introduced in [5], where a temporary cloud was formed by neighboring vehicles and/or roadside units (RSUs) through V2V and V2I communications. Early VC architectures supported simple tasks like intelligent traffic light control [6]. However, they lacked effective VC management mechanisms, making it difficult to discover suitable VCs and integrate resources across multiple VCs. As a result, vehicles struggled to dynamically discover and join appropriate VCs, and the system suffered from inefficient resource utilization and limited sensing coverage.

To support large-scale applications such as city-wide traffic flow monitoring, researchers proposed an architecture using a Center Cloud Manager (CCM) [2,7]. The CCM handles VC registration, enables VC discovery, etc. [6]. While effective for coordination, the CCM introduces a single point of failure—its unavailability prevents vehicles from accessing or updating VC information. To mitigate risks associated with a centralized CCM, edge computing concepts have been introduced in recent studies, where certain CCM functions are delegated to edge servers (e.g., RSUs) [8]. However, this architecture introduces new challenges. The lack of effective information-sharing mechanisms among different edge servers prevents vehicles from promptly obtaining the task statuses and resource availability of neighboring VCs, thereby hindering their optimal selection and participation in suitable VCs [9].

Given the challenges faced by existing VC architectures, blockchain technology offers promising features, including decentralization, transparency, and tamper resistance. Despite its potential, the integration of blockchain technology into VC management remains in its infancy, with limited research exploring how blockchains can enhance VC discovery, coordination, and resource sharing in a decentralized manner.

**Secure Establishment of VCs.** Over time, as the demand for security in VC environments has grown, various GKM schemes for the secure establishment of VCs have proposed—some tailored specifically for VCs and others originally designed for general group communication but potentially applicable in VC environments [10,11,12,13,14,15,16,17,18,19,20,21,22,23,24,25,26,27,28]. These schemes can be broadly categorized into two types: group key agreement (GKA)-based and group key distribution (GKD)-based schemes. GKA-based schemes are further divided into Traditional GKA (TGKA), Asymmetric GKA (AGKA), and Continuous GKA (CGKA) schemes.

GKA-based GKM schemes enable a group of entities (e.g., vehicles) to collaboratively establish a shared session key for secure communication. However, traditional GKA (TGKA) schemes, typically based on the Diffie–Hellman key exchange [10], require multiple rounds of interaction and assume that all members are simultaneously online and synchronized [11,12,13]. Furthermore, these schemes generally lack a formalized framework for continuous key updates within the protocol design, security model, and analysis. Their security assumptions are also relatively weak, often presuming fully trusted members without accounting for potential compromises of device states or intermediate computations. As a result, the applicability of TGKA schemes is significantly limited in asynchronous and dynamic environments such as VCs. To reduce interaction overhead, Non-Interactive GKA (NIGKA) schemes have been proposed, allowing users to compute a shared key without additional communication. However, existing constructions either only support small groups (e.g., the three-party scheme proposed by Joux [14]) or rely on expensive cryptographic primitives such as indistinguishability obfuscation (iO) [15], while often lacking essential properties like forward secrecy.

AGKA-based schemes enable a group of entities to negotiate a group public key and their respective private keys in a single round [16]. Several AGKA schemes have recently been proposed for VC environments [17,18,19,20]. While promising, these approaches face certain limitations. For instance, AGKA typically requires the maximum group size to be fixed during initialization, which can lead to unnecessary computational and storage overhead if the actual group size is smaller, limiting scalability in large groups. Furthermore, since VC communication occurs over public networks, open access to the group encryption key may expose the system to malicious requests or denial-of-service attempts. Finally, current AGKA schemes are often tightly coupled with bilinear pairing-based constructions, which may incur reactively high computational costs.

Recently, CGKA has been proposed to provide efficient key management for asynchronous, dynamic, and large-scale groups [21]. CGKA is also a potential candidate for secure communication in VC environments [22]. By leveraging a binary tree structure, CGKA achieves logarithmic communication and computational complexity, i.e., log(N) where *N* denotes the group size, for group membership changes such as members joining or leaving. However, CGKA lacks robust group management mechanisms, making it susceptible to insider attacks, and it depends on centralized servers for message delivery, which may limit its applicability in decentralized settings [23].

GKM schemes based on GKD typically rely on a trusted dealer (TD) to generate and distribute a shared group session key to all participating entities [24]. Although this approach avoids multiple rounds of interaction among entities, the reliance on a centralized TD introduces inherent limitations. First, the TD may become a single point of failure [25,26]. Second, due to the high mobility of vehicular clouds (VCs)—especially when the group is composed entirely of vehicles—it can be difficult to maintain a suitable and consistently available TD.

**Technological Readiness of Vehicular Sensors.** Modern vehicles have become highly integrated sensor platforms, providing rich data sources for VSNs. Commercially available sensors, such as LiDAR [29], 4D mmWave radar [30], and high-precision GPS sensors, can capture millisecond-level 3D information of the surrounding environment [1]. On the computational side, vehicles are equipped with high-performance onboard systems, such as the NVIDIA Orin [31], capable of efficiently processing large volumes of sensor data in real time. In terms of communication, many vehicles feature high-speed modules (e.g., 5.9 GHz C-V2X [32]), enabling low-latency and reliable data exchange. These commercial sensors and computing and communication modules allow for real-time sharing of traffic, road, and infrastructure information, supporting applications such as traffic flow optimization, signal control, and congestion warnings to improve road efficiency and safety. The security of these valuable data is increasingly important. The algorithms of the schemes proposed in this work (see Section 3 and Section 4) are fully compatible with existing commercial vehicle computing modules. At the same time, emerging devices, such as blockchain-enabled units, are beginning to appear in experimental and pilot deployments. Therefore, this work is forward-looking while remaining compatible with current VSN technologies.

### 1.2. Our Contribution

Due to the high level of mobility and dynamic nature of vehicular sensor networks (VSNs), traditional group key management (GKM) schemes are inadequate for establishing secure group channels for vehicular clouds (VCs) in such settings. To address this challenge, we propose a blockchain-based dynamic key management scheme, referred to as BcDKM. This scheme is built upon a slightly modified continuous group key agreement (CGKA) scheme—namely, CGKAwAM—and incorporates blockchain and smart contract mechanisms to address the core challenges identified in Section 1. The proposed scheme enables vehicles to establish secure group communication channels in VSNs and continuously update session keys to ensure communication security. The main contributions of this work are summarized as follows:

- We propose the CGKAwAM scheme, which extends CGKA scheme with a lightweight administrator-driven group member management mechanism. Unlike the standard CGKA scheme, CGKAwAM supports multiple administrators within a single VC. Each administrator is capable of independently processing user-initiated proposals (such as Join, Remove, and Update) in a single communication round.
- Based on CGKAwAM and blockchain technology, we design the BcDKM scheme, which provides several key advantages. First, it enables distributed VC discovery and management (see Section 2.2). RSUs act as blockchain nodes and use smart contracts to publish VC information, facilitating efficient matching and information sharing between vehicles and VCs. Additionally, multiple cloud administrators collaboratively manage each VC in a decentralized manner, reducing the risk of a single point of failure. Second, the scheme achieves round optimality and large-scale VC scalability (see Section 2.2). VC initialization and member join/leave operations can be completed asynchronously in a single communication round, without requiring all members to be online. Furthermore, the computational complexity grows logarithmically with the number of members, making the scheme suitable for large-scale deployment.
- We provide a rigorous formal security analysis for both the CGKAwAM and BcDKM schemes. Specifically, under a non-adaptive security model, we construct a game-based proof using the game-hopping technique, reducing the security of CGKAwAM to the CPA security of the underlying public key encryption scheme and the security of the pseudorandom generator. Our analysis shows that the proposed scheme satisfies multiple critical security properties, including key independence, forward secrecy, and post-compromise security. In addition, we analyze the security of the BcDKM scheme and show that it not only satisfies basic security requirements but also provides protection for vehicle privacy.
- We conduct a comprehensive evaluation of the proposed scheme through both theoretical analysis and simulation experiments. We also compare our scheme with several existing representative solutions. The results demonstrate that BcDKM achieves a balance between security and efficiency, confirming its practical feasibility.

The remainder of this paper is organized as follows. Section 2 presents the background, including the system model, design goals, and cryptographic tools. Section 3 describes the detailed design of the proposed CGKAwAM scheme and the associated smart contracts. Section 4 introduces the design of the BcDKM scheme. Section 5 provides a security analysis of the proposed schemes—namely, CGKAwAM and BcDKM. Section 6 evaluates the performance of the proposed schemes and presents the analysis results. Finally, Section 7 concludes the paper.

## 2. Background

### 2.1. System Model

**Figure 1:**
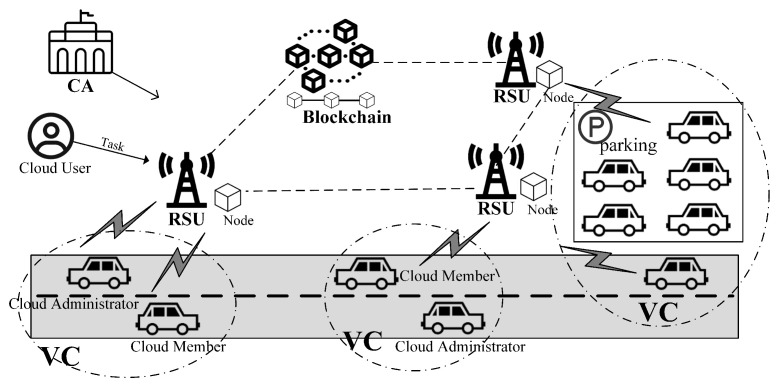
System model.

As shown in Figure 1, our system consists of the following entities:

- **Certificate Authority (CA)**: The CA is a trusted third party responsible for generating public system parameters and issuing digital certificates to vehicles and RSUs.
- **Vehicles**: Each vehicle is equipped with an onboard unit (OBU) that provides computing and communication capabilities and stores certificates and related secrets. A VC is formed by a group of vehicles and supports dynamic membership, allowing vehicles to join or leave the VC as needed. The vehicle that initiates the VC formation may act as the cloud administrator or delegate this responsibility to another vehicle, while the remaining participants serve as cloud members.
- **Roadside Unit (RSU)**: The RSU is located along the roadside; it communicates with vehicles within its coverage area, authenticates their identities, and publishes the VC information. Additionally, RSUs are assumed to function as part of the blockchain infrastructure, acting as blockchain nodes.
- **Blockchain (BC)**: The BC provides reliable data storage and supports automated execution via smart contracts. In our system, it is used to record VC-related information and assist VC administrators in managing the cloud.
- **Cloud Users (CUs)**: A CU refers to any authorized entity that accesses and utilizes the services provided by a VC. CUs can submit tasks to their selected VC for execution.

### 2.2. Threat Model and Design Goals

In our system, we assume that the CA is trusted and faithfully provides registration services to vehicles and RSUs. The blockchain is also assumed to be trusted, offering essential security properties such as resistance to 51% attacks [27]. However, both RSUs and vehicles are considered potentially malicious, as they are typically deployed in open environments and may be compromised by attackers. Additionally, we assume that the attacker has control over the wireless communication channel and is capable of eavesdropping on, injecting, transmitting, or modifying messages transmitted over the network.

Under this model, the security goals of our scheme are outlined as follows:

- **Vehicle privacy**: This property ensures that no one, except the CA, can learn the true identity of a cloud member (a vehicle) within a VC.
- **Known-key security**: This property ensures that even if an attacker obtains a session key of a cloud member within a particular VC, the security of other independent session keys remains uncompromised.
- **Forward secrecy**: This property ensures that if a vehicle is compromised by an attacker at a certain point in time, all session keys generated prior to the compromise remain confidential.
- **Post-compromise security**: This property ensures that if a vehicle is compromised at a certain point in time and the compromised vehicle subsequently performs a session key update that is not influenced by the attacker, then the updated session key remains confidential.

Our scheme also considers the following goals, in addition to the above security goals:

- **Distributed VC discovery**: This property enables a vehicle to efficiently discover existing VCs and their metadata via nearby RSUs, avoiding reliance on centralized platforms and mitigating single points of failure.
- **Distributed management**: This property ensures that each VC is managed by multiple cloud administrators. If one administrator becomes unavailable, others can still handle requests from vehicles inside or outside the VC (e.g., join and leave). This design mitigates insider threats and avoids single points of failure.
- **Round optimality**: This property ensures that group session key establishment and updates within a VC can be completed in a single round of communication, achieving optimal round complexity.
- **Large-scale VC scalability**: This property ensures efficient operation in VCs with many cloud members, as key management complexity grows logarithmically with group size.

**Remark** **1.** *It is worth noting that most existing VC-oriented GKM schemes define forward and backward security only against passive adversaries (i.e., eavesdropping capabilities) [12,26]. Forward Secrecy (not to be confused with our Forward secrecy) ensures that a passive adversary who knows a contiguous subset of old group keys cannot derive subsequent group keys, thereby preventing vehicles from accessing current communications after leaving the group. Backward Secrecy (not to be confused with our Post-compromise security) ensures that a passive adversary who knows a contiguous subset of group keys cannot derive preceding group keys, preventing newly joined vehicles from accessing past communications. In contrast, the forward security and post-compromise security offered by our scheme are stronger, as they not only resist passive eavesdropping but also withstand active adversaries (e.g., compromised vehicles) rather than being limited to passive threats.*

### 2.3. Building Blocks

In this section, we introduce several fundamental cryptographic tools that are employed in our system to achieve the desired goals. These building blocks include public key encryption (PKE), digital signature (DS), symmetric encryption (SE), pseudorandom generators (PRNGs), and PRG-based binary trees.

**Definition** **1** (PKE Scheme)**.** *A PKE scheme (*PKE = {PInit, PGen, PEnc, PDec}) *consists of the following algorithms:*

- *$\mathrm{PInit}(1^{\kappa })\to psp$: This algorithm accepts $1^{\kappa }$ and outputs the system parameters (psp).*
- *PGen(psp)→(sk,pk): This algorithm accepts psp and generates a private–public key pair (sk,pk).*
- *PEnc(pk,m)→cm: This algorithm accepts a plaintext (m) and a public key (pk) and outputs a ciphertext (cm).*
- *PDec(cm,sk)→m: This algorithm accepts a ciphertext (cm) and a secret key (sk) and outputs a plaintext (m).*

In our scheme, we rely on a PKE scheme that is secure against chosen plaintext attacks (CPAs), a widely accepted security notion in modern cryptography. Let ${\mathrm{Adv}}_{A}^{\mathrm{CPA}}$ denote the adversary’s advantage in breaking the PKE scheme; the corresponding formal security definition is given in Appendix A.

**Definition** **2** (DS Scheme)**.** *A digital signature scheme (DS = {DInit, DGen, DSign, DVer}) consists of the following algorithms:*

- *$\mathrm{DGen}\left(1^{\kappa }\right)\to \left(sk,pk\right)$: This algorithm takes $1^{\kappa }$ as input and outputs a private–public key pair (sk,pk).*
- *DSign(sk,m)→σ: This algorithm takes sk and a message (m) as input and outputs a signature (σ).*
- *DVer(pk,m,σ)→{0,1}: This algorithm takes pk, a message (m), and a signature (σ) as input and outputs 1 if the signature is valid or 0 otherwise.*

In our scheme, we rely on a digital signature scheme that is existentially unforgeable under chosen message attacks (EUF-CMA).

**Definition** **3** (SE Scheme)**.** *A symmetric encryption scheme (SE={SGen,SEnc,SDec}) consists of the following algorithms:*

- *$\mathrm{SGen}(1^{\kappa })\to k$: This algorithm takes $1^{\kappa }$ as input and outputs a secret key (k).*
- *SEnc(k,m)→c: This algorithm takes a secret key (k) and a message (m) as input and outputs a ciphertext (c).*
- *SDec(k,c)→m: This algorithm takes k and a ciphertext (c) as input and outputs a message (m).*

In our scheme, we rely on a symmetric encryption scheme that is semantically secure.

**Definition** **4** (RPG)**.** *A PRG is a function, i.e., $H_{\mathrm{prg}}:R\to K\times R$, where R denotes the random seed space and K denotes the secret key space.*

Let ${\mathrm{Adv}}_{A}^{\mathrm{PRG}}$ denote the adversary’s advantage in breaking the PRG; the corresponding formal security definition is given in Appendix A.

We define a helper function ($H_{\mathrm{prg}}^{d}\left(x\right)$) by iteratively invoking the PRG ($H_{\mathrm{prg}}$) for *d* rounds, starting from an initial seed (x∈R). Formally, let $x_{1}=x$. For each i∈{1,…,d}, we compute $\left(sk_{i},x_{i+1}\right)\leftarrow H_{\mathrm{prg}}\left(x_{i}\right)$. The output of the helper function is defined as $H_{\mathrm{prg}}^{d}\left(x\right)=sk_{d}$, i.e., only the secret key ($sk_{d}$) obtained in the *d*-th iteration is returned as the final result.

**Definition** **5** (PRG-based Binary Tree)**.** *A PRG-based binary tree (PBT) is a full binary tree used for key derivation. A PBT consists of Y nodes, denoted as ${\left\{A_{j}\right\}}_{j=1}^{Y}$, and has a height of $h=\lfloor \log_{2}\left(Y\right)\rfloor +1$. Specifically, let $A_{1}$ be the root node. The leaf nodes are represented as ${\left\{A_{2^{h-1}+z}\right\}}_{0\leq z\leq 2^{h-1}-1}$, while the remaining internal (non-leaf) nodes are represented as ${\left\{A_{z+1}\right\}}_{1\leq z<2^{h-1}-1}$. Each node ($A_{j}\in {\left\{A_{j}\right\}}_{j=1}^{Y}$) stores a unique tuple in the form of (sk,pk,he,e,sv), where sv denotes a secret value and sk is a secret key derived from sv using a PRG. The public key (pk) is derived from sk using a public key derivation algorithm ($pk_{sk}\leftarrow \mathrm{DerivePK}\left(sk\right)$). In our scheme, pk is derived from sk using the same method as in the PGen(psp) of a PKE scheme, making the resulting key pairs fully compatible with the corresponding PKE scheme. The value of he indicates the height of the node in the tree (e.g., each leaf node is assigned he=1, while the root node is assigned he=h), and e indicates whether a node is a leaf (with e=1 denoting leaf nodes and e=0 denoting non-leaf nodes). The values in each tuple are initialized using the algorithm expressed as ${\mathrm{PBT}}_{\mathrm{Init}}\left({\left\{a_{i}\right\}}_{i=1}^{n}\right)\to {\left\{A_{j}\right\}}_{j=1}^{Y}$ (defined below) and updated using the algorithm expressed as ${\mathrm{PBT}}_{\mathrm{Upd}}\left({\left\{A_{j}\right\}}_{j=1}^{Y},I,ra\right)\to {\left\{A_{j}\right\}}_{j=1}^{Y}$ (as defined below). The size of a PBT can also be expanded to include additional nodes using the algorithm expressed as ${\mathrm{PBT}}_{\mathrm{Ext}}\left({\left\{A_{j}\right\}}_{j=1}^{Y}\right)\to {\left\{A_{k}^{\prime }\right\}}_{k=1}^{2Y+1}$. A formal description of the ${\mathrm{PBT}}_{\mathrm{Ext}}$ algorithm is provided in Appendix B (see Algorithm A2).*

The algorithm expressed as ${\mathrm{PBT}}_{\mathrm{Init}}\left({\left\{a_{i}\right\}}_{i=1}^{n}\right)\to {\left\{A_{j}\right\}}_{j=1}^{Y}$ is used to initialize a PBT. In our scheme, it is executed by a cloud administrator and is used to initialize a VC (see Section 4.5 for VC initialization). Given a set of secret values (${\left\{a_{i}\right\}}_{i=1}^{n}$, as chosen by a cloud administrator), the algorithm outputs a PBT ${\left\{A_{j}\right\}}_{j=1}^{Y}$ with a height of $h=\lceil \log_{2}\left(n\right)\rceil +1$ and $Y=2^{\lceil \log_{2}\left(n\right)\rceil +1}-1$. A formal description of the ${\mathrm{PBT}}_{\mathrm{Init}}$ algorithm is provided in algorithm is provided in Appendix B (see Algorithm A1).

In our scheme, the algorithm expressed as ${\mathrm{PBT}}_{\mathrm{Upd}}\left({\left\{A_{j}\right\}}_{j=1}^{Y},I,ra\right)\to {\left\{A_{j}\right\}}_{j=1}^{Y}$ is executed by a cloud administrator or a cloud member to update the nodes along the path from the leaf node at position *I* (where *I* denotes the index of a node in the leaf layer) to the root of the PBT (${\left\{A_{j}\right\}}_{j=1}^{Y}$) using a random value (ra). Given a PBT (${\left\{A_{j}\right\}}_{j=1}^{Y}$), a leaf position (*I*), and a random value (ra) selected by the cloud administrator or a cloud member, the algorithm outputs an updated PBT (${\left\{A_{j}\right\}}_{j=1}^{Y}$). A formal description of the ${\mathrm{PBT}}_{\mathrm{Upd}}$ algorithmis provided in Appendix B (see Algorithm A3).

In our scheme, the PBT is utilized to support the continuous and dynamic updating of session keys in a VC. To achieve these goals, we define several helper algorithms to retrieve paths and associated nodes from the PBT.

- The algorithm expressed as ${\mathrm{PBT}}_{\mathrm{Path}}\left({\left\{A_{j}\right\}}_{j=1}^{Y},I\right)\to {\left\{A_{i}\right\}}_{i=1}^{Z}$, where $Z=\lfloor \log_{2}\left(Y\right)\rfloor +1$, is used to retrieve the sequence of nodes along the path from the *I*-th leaf node to the root of the given PBT ${\left\{A_{j}\right\}}_{j=1}^{Y}$. It is typically executed by the cloud administrator or a cloud member. Given a PBT (${\left\{A_{j}\right\}}_{j=1}^{Y}$) and position (*I*) selected by the cloud administrator or a cloud member, the algorithm returns a path (${\left\{A_{i}\right\}}_{i=1}^{Z}$) that consists of direct references to the original PBT nodes so that any modification of a node in the path immediately affects the original PBT nodes.
- The algorithm expressed as ${\mathrm{PBT}}_{\mathrm{Epath}}\left({\left\{A_{j}\right\}}_{j=1}^{Y},{\left\{A_{i}\right\}}_{i=1}^{Z}\right)\to {\left\{A_{j}^{\prime }\right\}}_{j=1}^{Y}$, where $Z=\lfloor \log_{2}\left(Y\right)\rfloor +1$, is used to generate a modified copy of a given PBT (${\left\{A_{j}\right\}}_{j=1}^{Y}$), with all sensitive information (i.e., private key and secret value) outside the specified path (${\left\{A_{i}\right\}}_{i=1}^{Z}$) masked. It is typically executed by the cloud administrator. Given a PBT (${\left\{A_{j}\right\}}_{j=1}^{Y}$) and a path (${\left\{A_{i}\right\}}_{i=1}^{Z}$) within the PBT selected by the cloud administrator, the algorithm returns a duplicate PBT (${\left\{A_{j}^{\prime }\right\}}_{j=1}^{Y}$), in which the private keys and secret values of all nodes not included in the path (${\left\{A_{i}\right\}}_{i=1}^{Z}$) are set to null.
- The algorithm expressed as ${\mathrm{PBT}}_{\mathrm{RCA}}\left({\left\{A_{j}\right\}}_{j=1}^{Y},A_{k},A_{k^{\prime }}\right)\to \left\{A_{k^{\prime \prime }},\ldots ,A_{1}\right\}$ is used to obtain the set of common ancestor nodes shared by $A_{k}$ and $A_{k^{\prime }}$ in the tree expressed as ${\left\{A_{i}\right\}}_{i=1}^{Y}$, ranging from their lowest common ancestor ($A_{k^{\prime \prime }}$) up to the root node ($A_{1}$).

## 3. Building Blocks

In this section, we first design a continuous group key agreement scheme with an administrative mechanism (CGKAwAM), which supports multiple administrators and provides a continuous key stream for dynamic groups. We next introduce two designed smart contracts: VIDSC and VCSC. Based on CGKAwAM and the proposed contracts, we then construct a blockchain-based dynamic key management scheme tailored for vehicle cloud (VC) systems (see Section 4).

### 3.1. CGKA with Administrator Mechanism (CGKAwAM)

A CGKAwAM (SetUp,KeyGen,Create,Proposal,Commit,Process) comprises the following algorithms:

- **SetUp(κ)→csp**: This algorithm is executed by a trusted authority (e.g., certification authority) to generate system parameters. Upon input of a security parameter (κ), it outputs a common system parameter (csp). The trusted authority proceeds as follows:
  1. Select a public key encryption scheme (PKE={PInit,PGen,PEnc,PDec}). Invoke $\mathrm{PInit}(1^{\kappa })$ to obtain psp (see Definition 1).
  2. Choose a secure PRG ($H_{\mathrm{prg}}:R\to K\times R$; see Definition A2).
  3. Publish the common system parameter (csp=(PKE, $H_{PRG}$, psp)).
- **KeyGen$\left(csp,u_{i}\right)\to KP_{u_{i}}$**: This algorithm is executed by a user with identifier $u_{i}$ to generate their own key pair. Upon input of csp and $u_{i}$, it outputs a public–private key pair ($KP_{u_{i}}=\left(sk_{u_{i}},pk_{u_{i}}\right)$). Internally, it invokes PGen(psp) to generate the key pair ($KP_{u_{i}}$).
- **Create**$(csp,G,u_{1})\to$ ($\gamma_{u_{1}}^{gid}$, CM): This algorithm is executed by user $u_{1}$, acting as the group administrator, to initiate a new group. Upon input of csp, an identifier set ($G=\{u_{1},\ldots ,u_{n}\}$ with n>1), and an identifier ($u_{1}\in G$), it outputs the administrator’s local state ($\gamma_{u_{1}}^{gid}$, where gid is the newly assigned group identifier), and a set of control messages (CM) is used to invite the remaining members in *G* to join the group. The state encapsulates the user’s status in the group, including the session key, the list of group administrators, the list of group members, public parameters, and so on. Furthermore, suppose that each user $u_{i}\in G$ holds a key pair $\left(sk_{u_{i}},\;pk_{u_{i}}\right)$. $u_{1}$ initializes a group as follows:
  1. Set the group identifier ($gid=H_{PRG}\left(Str_{G}\right)$, where $Str_{G}=u_{1}\left||\ldots |\right|u_{n}\left|\right|ts$ ts is a timestamp).
  2. Select *s* users from *G* to serve as administrators, forming the set expressed as Adm. The remaining users form the set expressed as Mem=G∖Adm.
  3. Assign each user ($u_{i}\in G$) a unique random number ($a_{i}\in R$). This yields the set expressed as $\left\{a_{1},\ldots ,a_{n}\right\}$.
  4. Invoke ${\mathrm{PBT}}_{\mathrm{Init}}\left({\left\{a_{i}\right\}}_{i=1}^{n}\right)$ to construct a PBT ${\left\{A_{j}\right\}}_{j=1}^{Y}$, where $Y=2^{h}-1$ and $h=\lceil \log_{2}\left(n\right)\rceil +1$.
  5. Compute the session key as $rsk=H_{\mathrm{prg}}\left(\bar{A_{1}}\right)$, where $\bar{A_{1}}=sk\;∥\;pk\;∥\;h\;∥\;e\;∥\;sv$ is the serialized string of the tuple stored at the root node $A_{1}$; see Definition 5.
  6. Initialize a mapping array (Map[]) of length $\mathrm{len}=2^{h-1}$ to associate each PBT leaf node with a user from *G*. Set $\mathrm{Map}\left[i\right]\leftarrow u_{i}$ for 1≤i≤n and Map[i]←null for n<i≤len.
  7. For each user with identifier $u_{z}$ in $G\setminus \{V_{1}\}$, generate a control message as follows:
    – If $u_{z}\in \mathrm{Adm}$, do the following:
      (a) Invoke $\mathrm{PEnc}(pk_{u_{z}},{\left\{A_{j}\right\}}_{j=1}^{Y}\;\|\;ip\;\|\;n\;\|\;\mathrm{Map}\;\|\;len)$ to obtain the ciphertext ($cm_{z}$), where ip denotes the position assigned to user $u_{z}$ in the PBT (i.e., the ip-th leaf node such that $\mathrm{Map}\left[ip\right]=u_{z}$).
      (b) Add the control message ($cm_{z}=(\mathrm{Join}\;\|\;cp_{z}\;\|\;u_{1}\;\|\;{\mathrm{sc}}_{vid}\;\|\;\mathrm{Adm}\;\|\;\mathrm{Mem})$) to set CM.
    – Otherwise, do the following:
      (a) Invoke ${\mathrm{PBT}}_{\mathrm{Path}}\left({\left\{A_{j}\right\}}_{j=1}^{Y},ip\right)$ to obtain a path (${\left\{A_{j^{\prime }}\right\}}_{j^{\prime }=1}^{L}$, where $L=\lfloor \log_{2}\left(Y\right)\rfloor +1$, $\mathrm{Map}\left[ip\right]=u_{z})$.
      (b) Invoke ${\mathrm{PBT}}_{\mathrm{Epath}}\left({\left\{A_{j}\right\}}_{j=1}^{Y},{\left\{A_{j^{\prime }}\right\}}_{j^{\prime }=1}^{L}\right)$ to obtain a PBT (${\left\{A_{j}^{\prime }\right\}}_{j=1}^{Y}$).
      (c) Invoke $\mathrm{PEnc}(pk_{u_{z}}$, ${\left\{A_{j}^{\prime }\right\}}_{j=1}^{Y}\;\|\;ip\;\|\;n\;\|\;\mathrm{Map}\;\|\;len)$ to obtain $cm_{z}$.
      (d) Add the control message ($cm_{z}=(\mathrm{Join}\;\|\;cp_{z}\;\|\;u_{1}\;\|\;\mathrm{Adm}\;\|\;\mathrm{Mem})$) to set CM.
  8. Set the state as $\gamma_{u_{1}}^{gid}=\left(\mathrm{Role},\mathrm{Adm},\mathrm{Mem},\mathrm{Map},\mathrm{len},n,{\left\{A_{j}\right\}}_{j=1}^{Y},rsk,\mathrm{index}\right)$, where Role=adm indicates that $u_{1}$ is designated as the administrator and index=1 indicates that $u_{1}$ is mapped to the first leaf node of the PBT, i.e., $\mathrm{Map}\left[\mathrm{index}\right]=u_{1}$.
  9. Send each control message in set CM to the corresponding user.
- **Proposal$(csp,gid,u_{i},u_{a},prop,txt)\to PM$**: This algorithm is executed by user $u_{i}$ to generate a proposal message (PM) intended for the group administrator ($u_{a}$) within the gid group, with the purpose of requesting a group membership change (e.g., join or leave) or a session key update. Upon input of $csp,gid,u_{i},u_{a}$ and a proposal type (prop∈{ADD,REM,UPD}), it outputs the proposal message (PM). The prop parameter specifies the purpose of the proposal: adding a member, removing a member, or updating the session key. The txt field represents the payload associated with the proposal. We present three example proposal messages used in our scheme as follows.
  – $PM=(\mathrm{gid}\;\|\;\mathrm{ADD}\;\|\;u_{a}\;\|\;u_{i}\;\|\;\mathrm{txt})$: A join request from an external user ($u_{i}$), asking administrator $u_{a}$ to add it to group gid, where txt=NULL.
  – $PM=(\mathrm{gid}\;\|\;\mathrm{REM}\;\|\;u_{a}\;\|\;u_{i}\;\|\;\mathrm{txt})$: A removal request by current group member $u_{i}$, requesting to leave or be removed from the gid group, where txt=NULL.
  – $PM=(\mathrm{gid}\;\|\;\mathrm{UPD}\;\|\;u_{a}\;\|\;u_{i}\;\|\;\mathrm{txt})$: A key update request by current group member $u_{i}$, asking $u_{a}$ to update the group session key, with $\mathrm{txt}=\mathrm{PEnc}(pk_{u_{a}},sd)$, where $pk_{u_{a}}$ is the public key of $u_{a}$ and sd is randomly selected by $u_{i}$.
  User $u_{i}$ sends the proposal message (PM) to the administrator ($u_{a}$).
- **Commit**$\left(csp,\gamma_{u_{a}}^{gid},PM\right)\to \left({\gamma^{\prime }}_{u_{a}}^{gid},\mathrm{CM}\right)$: This algorithm is executed by user $u_{a}$, who serves as the administrator of the gid group. It is used to process a proposal message (PM) submitted by another user. Upon input of $csp,\gamma_{u_{a}}^{gid},PM$, it outputs the updated state (${\gamma^{\prime }}_{u_{a}}^{gid}$) and a set of control messages (CM), which are used by the other group members to update their local states. Suppose $u_{a}$ in a group (gid) has a local state of $\gamma_{u_{a}}^{gid}$ = ($\mathrm{Role},\mathrm{Adm},\mathrm{Mem},\mathrm{Map},len,n,{\left\{A_{j}\right\}}_{j=1}^{Y},rsk$, index). Suppose $PM=(gid\;\|\;prop\;\|\;u_{a}\;\|\;u_{i}\;\|\;txt)$. $u_{a}$ then processes the PM as follows:
  – If prop=ADD, do the following:
    1. Search the Map array for an index (ip) such that Map[ip]=⌀. If no such index exists (i.e., all current leaf nodes are occupied), invoke the helper function (${\mathrm{PBT}}_{\mathrm{Ext}}\left({\left\{A_{j}\right\}}_{j=1}^{Y}\right)$) to extend the PBT, and search again for an empty index (ip). Once an empty index is found, set $\mathrm{Map}\left[ip\right]=u_{i}$, n=n+1, and add $u_{i}$ to the the Mem set.
    2. Select a random value ($a_{r}$) for user $u_{i}$.
    3. Generate the corresponding control messages for user $u_{i}$ as follows:
      (a) Invoke ${\mathrm{PBT}}_{\mathrm{Upd}}\left({\left\{A_{j}\right\}}_{j=1}^{Y},ip,a_{r}\right)$ to update the path in the PBT (${\left\{A_{j}\right\}}_{j=1}^{Y}$).
      (b) Invoke ${\mathrm{PBT}}_{\mathrm{Path}}\left({\left\{A_{j}\right\}}_{j=1}^{Y},ip\right)$ to obtain the updated path (${\left\{A_{j^{\prime }}\right\}}_{j^{\prime }=1}^{L=\lceil \log_{2}\left(n\right)\rceil +1}$).
      (c) Invoke ${\mathrm{PBT}}_{\mathrm{Epath}}\left({\left\{A_{j}\right\}}_{j=1}^{Y},{\left\{A_{j^{\prime }}\right\}}_{j^{\prime }=1}^{L}\right)$ to obtain ${\left\{A_{j}^{\prime }\right\}}_{j=1}^{Y}$.
      (d) Invoke $\mathrm{PEnc}(pk_{u_{i}}$, ${\left\{A_{j}^{\prime }\right\}}_{j=1}^{Y}\;\|\;ip\;\|\;n\;\|\;\mathrm{Map}\;\|\;len)$ to obtain $cp_{i}$.
      (e) Add the message expressed as $cm_{i}=(\mathrm{Join}\;\|\;cp_{i}\;\|\;u_{a}\;\|\;u_{i}\;\|\;\mathrm{Adm}\;\|\;\mathrm{Mem})$ to set CM.
    4. Generate the corresponding control messages for administrators ($u_{z}\in \mathrm{Adm}\setminus \left\{u_{a}\right\}$) as follows:
      (a) Invoke $\mathrm{PEnc}(pk_{u_{z}},{\left\{A_{j}\right\}}_{j=1}^{Y},len,n,\mathrm{Map})$ to obtain the ciphertext ($cp_{z}$).
      (b) Add the message expressed as $cm_{z}=(\mathrm{Admin}\;\|\;cp_{z}\;\|\;u_{a}\;\|\;u_{i}\;\|\;\mathrm{Adm}\;\|\;\mathrm{Mem})$ to set CM.
    5. Generate the corresponding control messages for the other group members (i.e., $\left\{\mathrm{Mem}\setminus u_{i}\right\}$) as follows:
      (a) Invoke ${\mathrm{PBT}}_{\mathrm{Path}}\left({\left\{A_{j}\right\}}_{j=1}^{Y},ip\right)$ to obtain the path expressed as
        ${\left\{A_{j^{\prime \prime \prime }}\right\}}_{j^{\prime \prime \prime }=1}^{L=\lceil \log_{2}\left(n\right)\rceil +1}$.
      (b) For each node on the path expressed as $A_{k}\in {\left\{A_{j^{\prime \prime \prime }}\right\}}_{j^{\prime \prime \prime }=1}^{L}$, do the following:
        i. If node $A_{k}$ has a non-empty sibling node ($A_{k^{\prime }}$) in the PBT (${\left\{A_{j}\right\}}_{j=1}^{Y}$, i.e., $A_{k}$ and $A_{k^{\prime }}$ share the same direct parent node, where $k^{\prime }=k+1$ or $k^{\prime }=k-1$), then retrieve the tuple expressed as (sk,pk,he,e,sv) from node $A_{k^{\prime }}$ and invoke ${\mathrm{PBT}}_{\mathrm{RCA}}\left({\left\{A_{j}\right\}}_{j=1}^{Y},A_{k},A_{k^{\prime }}\right)$ to obtain the node set expressed as $\left\{A_{k^{\prime \prime }},\ldots ,A_{1}\right\}$, which represents the sequence of common ancestor nodes of $A_{k}$ and $A_{k^{\prime }}$, up to the root node $A_{1}$. The total number of nodes in this set is he. Otherwise, continue the loop.
        ii. Invoke $\mathrm{PEnc}(pk,\left\{A_{k^{\prime \prime }},\ldots ,A_{1}\right\}\;\|\;L_{PK})$ to obtain the ciphertext $cp_{k}$, where $L_{PK}$ is a list of public keys corresponding to those stored in the node along the path expressed as ${\left\{A_{j^{\prime \prime \prime }}\right\}}_{j^{\prime \prime \prime }=1}^{L}$.
        iii. For each non-administrator user ($u_{v}\in \mathrm{Mem}\setminus \left\{u_{i}\right\}$) whose identifier is stored in the array (Map[q], where *q* satisfies $k\cdot 2^{he-1}\leq q\leq \left(k+1\right)\cdot 2^{he-1}-1$), set the control message as $cm_{v}=(prop\;\|\;cp_{k}\;\|\;u_{a}\;\|\;u_{v}\;\|\;u_{i}\;\|\;ip)$. Note that the control messages for these users are identical, except for the recipient identifier ($u_{v}$). Add each message ($cm_{v}$) to the control message set (CM).
  – If prop=REM, do the following:
    1. Search the array (Map) for an index (ip) such that $\mathrm{Map}\left[ip\right]=u_{i}$. Then, set Map[ip]=⌀, n=n−1 and remove $u_{i}$ from the Mem set.
    2. Select a random value ($a_{r}$).
    3. Invoke ${\mathrm{PBT}}_{\mathrm{Upd}}\left({\left\{A_{j}\right\}}_{j=1}^{Y},ip,a_{r}\right)$ to update the path in the PBT (${\left\{A_{j}\right\}}_{j=1}^{Y}$).
    4. Generate the corresponding control messages for the other administrators (i.e., $\mathrm{Adm}\setminus \{u_{a}\}$) in the same way as Step 4 under the condition of prop=ADD in this algorithm.
    5. Generate the corresponding control messages for the other group members (i.e., $\mathrm{Mem}\setminus \{u_{i}\}$) in the same way as Step 5 under the condition of prop=ADD in this algorithm.
  – If prop=UPD, do the following:
    1. Search the array (Map) for an index (ip) such that $\mathrm{Map}\left[ip\right]=u_{i}$.
    2. Obtain the tuple expressed as (sk,pk,he,e) from the node leaf ($A_{\frac{Y+1}{2}-1+ip}$).
    3. Compute $a_{r}=\mathrm{PDec}\left(sk_{u_{a}},txt\right)$, where $sk_{u_{a}}$ is the private key stored in the node expressed as $A_{\frac{Y+1}{2}-1+index}$.
    4. Invoke ${\mathrm{PBT}}_{\mathrm{Upd}}\left({\left\{A_{j}\right\}}_{j=1}^{Y},ip,a_{r}\right)$ to update the path in the PBT (${\left\{A_{j}\right\}}_{j=1}^{Y}$).
    5. Generate the corresponding control message for $u_{i}$, and proceed as follows:
      (a) Invoke ${\mathrm{PBT}}_{\mathrm{Path}}\left({\left\{A_{j}\right\}}_{j=1}^{Y},ip\right)$ to obtain a path (${\left\{A_{j^{\prime \prime }}\right\}}_{j^{\prime \prime }=1}^{L=\lceil \log_{2}\left(Y\right)\rceil +1}$).
      (b) Set the cipher to $cp_{i}=NULL$.
      (c) Add the control message ($cm_{i}=(prop\;\|\;cp_{i}\;\|\;u_{a}\;\|\;u_{i}\;\|\;ip)$) to set CM.
    6. Generate the corresponding control messages for the other administrators (i.e., $\mathrm{Adm}\setminus \{u_{a}\}$) in the same way as Step 4 under the condition of prop=ADD in this algorithm.
    7. Generate the corresponding control messages for the other group members (i.e., $\mathrm{Mem}\setminus \{u_{i}\}$) in the same way as Step 5 under the condition of prop=ADD in this algorithm.
  – Compute the updated session key as $rsk^{\prime }=H_{\mathrm{prg}}\left(rsk\;\|\;\bar{A_{1}}\right)$. Note that $H_{prg}\left(rsk\;\|\;\bar{A_{1}}\right)$ denotes the PRG computation with the concatenation of the session key (rsk) and the tuple stored in node $A_{1}$.
  – Update the state ($\gamma_{u_{a}}^{gid}$ = (Role,Adm,Mem,Map, len, *n*, ${\left\{A_{j}\right\}}_{j=1}^{Y}$, $rsk^{\prime }$, index)).
- $\mathrm{Process}\left(gid,u_{i},\gamma_{u_{i}}^{gid},cm\right)\to \left({\gamma^{\prime }}_{ID_{u}}^{gid}\right)$: This algorithm is executed by user $u_{i}$, who is a member of the gid group. It is used to process a control message (cm) sent by the group administrator within the gid group for the purpose of updating the user’s local session state. Upon input of gid, $u_{i}$, $\gamma_{u_{i}}^{gid}$, and cm, it outputs the updated state (${\gamma^{\prime }}_{u_{a}}^{gid}$). If user $u_{i}$ receives the control message (cm) for the first time, then inputs gid and $\gamma_{ID}^{gid}$ are set as *⌀*. Suppose that $u_{i}$ receives the control message (cm) from administrator $u_{a}$ within the gid group and $u_{i}$ is associated with a key pair $\left(sk_{u_{i}},pk_{u_{i}}\right)$. $u_{i}$ then processes cm as follows:
  – A cm format of $\left(\mathrm{Join}\;\|\;cp\;\|\;u_{a}\;\|\;u_{i}\;\|\;\mathrm{Adm}\;\|\;\mathrm{Mem}\right)$ indicates that $u_{i}$ is joining group gid for the first time, then proceeds as follows:
    1. Invoke $\mathrm{DEnc}(sk_{u_{i}},cp)$ to obtain $\left({\left\{A_{j}\right\}}_{j=1}^{Y}\;\|\;ip\;\|\;n\;\|\;\mathrm{Map}\;\|\;len\right)$, where $\mathrm{Map}\left[ip\right]=u_{i}$.
    2. Compute the session key as rsk = $H_{\mathrm{prg}}\left(\bar{A_{1}}\right)$.
    3. If $u_{i}\in \mathrm{Adm}$, then set Role=adm; otherwise, set Role=mem.
    4. Set state $\gamma_{u_{i}}^{gid}$ = $\left(\mathrm{Role},\mathrm{Adm},\mathrm{Mem},\mathrm{Map},len,n,{\left\{A_{j}\right\}}_{j=1}^{Y},rsk,ip\right)$.
  – Otherwise, a cm format of $\left(\mathrm{Admin}\;\|\;cp\;\|\;u_{a}\;\|\;u_{i}\;\|\;\mathrm{Adm}\;\|\;{\mathrm{Mem}}^{\prime }\right)$ indicates that $u_{i}$ is a cloud administrator within the gid group. Suppose $u_{i}$ possesses a local state of $\gamma_{u_{i}}^{gid}$ = $\left(\mathrm{Role},\mathrm{Adm},\mathrm{Mem},\mathrm{Map},len,n,{\left\{A_{j}\right\}}_{j=1}^{Y},rsk,ip\right)$. Then, $u_{i}$ proceeds as follows:
    1. Invoke $\mathrm{DEnc}(sk_{u_{i}},cp)$ to obtain (${\left\{A_{j}^{\prime }\right\}}_{j=1}^{Y},len^{\prime },n^{\prime },\mathrm{Map}’$).
    2. Compute the session key as $rsk^{\prime }$ = $H_{PRG}\left(rsk\;\|\;\bar{A_{1}}\right)$.
    3. Set state $\gamma_{u_{i}}^{gid}$ = ($\mathrm{Role},\mathrm{Adm},{\mathrm{Mem}}^{\prime },\mathrm{Map}’,len^{\prime },n^{\prime },{\left\{A_{j}^{\prime }\right\}}_{j=1}^{Y},rsk^{\prime },ip$).
  – Otherwise, the control message (cm) originates from the gid group, which $u_{i}$ has already joined. Suppose cm has a format of $\left(prop\;\|\;cm\;\|\;u_{a}\;\|\;u_{i}\;\|\;u_{i}^{\prime }\;\|\;ip\right)$ and user $u_{i}$ possesses a local state of $\gamma_{u_{i}}^{gid}$ = (Role,Adm,Mem,Map,len,n, ${\left\{A_{j}\right\}}_{j=1}^{Y}$, rsk, index). $u_{i}$ does the following:
    * If prop∈{ADD/UPD/REM} and $u_{i}\neq u_{i}^{\prime }$, another member has either joined the group, updated the group session key, or exited. $u_{i}$ proceeds as follows:
      1. If prop=ADD, then set $\mathrm{Map}\left[ip\right]=u_{i}^{\prime }$, n=n+1 and add $u_{i}^{\prime }$ to the Mem set.
      2. If prop=REM, then set Map[ip]=⌀, n=n−1 and remove $u_{i}^{\prime }$ from the Mem set.
      3. Invoke ${\mathrm{PBT}}_{\mathrm{RCA}}\left({\left\{A_{j}\right\}}_{j=1}^{Y},A_{\frac{Y+1}{2}-1+ip},A_{\frac{Y+1}{2}-1+index}\right)$ to obtain the node set expressed as $\left\{A_{lca},\ldots ,A_{1}\right\}$, which represents the sequence of common ancestor nodes of $A_{\frac{Y+1}{2}-1+ip}$ and $A_{\frac{Y+1}{2}-1+index}$, up to the root node ($A_{1}$). $A_{lca}$ is the lowest common ancestor.
      4. Obtain the tuple expressed as (sk,pk,he,e,sv) from the child node of $A_{lca}$, which is also an ancestor of $A_{\frac{Y+1}{2}-1+\mathrm{index}}$.
      5. Invoke PDec(sk, cm) to obtain $\left(\left\{A_{k^{\prime \prime }},\ldots ,A_{1}\right\}\;\|\;L_{pk}\right)$.
      6. Replace the nodes in the set expressed as $\left\{A_{lca},\ldots ,A_{1}\right\}$, which belong to the PBT ${\left\{A_{j}\right\}}_{j=1}^{Y}$, with the corresponding nodes in the set expressed as $\left\{A_{k^{\prime \prime }},\ldots ,A_{1}\right\}$.
      7. Replace the public keys in the tuples stored in the nodes along the path from the leaf node ($A_{\frac{Y+1}{2}-1+\mathrm{index}}$) to the root node ($A_{1}$) with the corresponding public keys in list $L_{pk}$.
      8. Compute the updated session key as $rsk^{\prime }$ = $H_{PRG}\left(rsk|\right|\bar{A_{1}})$.
      9. Set state $\gamma_{u_{i}}^{gid}$ = $\left(\mathrm{Role},\mathrm{Adm},\mathrm{Mem},\mathrm{Map},len,n,{\left\{A_{j}\right\}}_{j=1}^{Y},rsk^{\prime },index\right)$.
    * Otherwise, the received control message (cm) is the administrator’s response to the proposal message submitted by $u_{i}$, where $PM=(\mathrm{gid}\;\|\;\mathrm{UPD}\;\|\;u_{a}\;\|\;u_{i}\;\|\;\mathrm{txt})$, with $\mathrm{txt}=\mathrm{PEnc}(pk_{u_{a}},sd)$ and sd being a random value chosen by $u_{i}$. The message is processed as follows:
      1. Invoke ${\mathrm{PBT}}_{\mathrm{Upd}}\left({\left\{A_{j}\right\}}_{j=1}^{Y},index,sd\right)$ to update the corresponding path.
      2. Compute the updated session key as $rsk^{\prime }$ = $H_{PRG}\left(rsk|\right|\bar{A_{1}})$.
      3. Set state $\gamma_{u_{i}}^{gid}$ = $\left(\mathrm{Role},\mathrm{Adm},\mathrm{Mem},\mathrm{Map},len,n,{\left\{A_{j}\right\}}_{j=1}^{Y},rsk^{\prime },index\right)$.

### 3.2. Design of Smart Contract

A smart contract is a self-executing program deployed on the blockchain, typically written in a Turing-complete programming language such as Solidity, Vyper, or DAML. It consists of variables for data storage and methods that can be invoked by entities to manipulate these variables. Each smart contract has a unique (public) address on the blockchain. All state changes resulting from method invocations are ultimately recorded on all blockchain nodes through the underlying consensus mechanism.

#### 3.2.1. Vehicle Identity Smart Contract (VIDSC)

In our scheme, each RSU deploys and maintains a VIDSC to securely record and manage information about authenticated vehicles that intend to join the VC. The VIDSC is formally defined in Appendix C (see Algorithm A4).

Specifically, the VIDSC contract declares a data structure named VID to store essential vehicle information. This structure contains two fields: CredID, representing the vehicle’s (anonymous) authentication certificate, and Source, describing the vehicle’s sensing or computational capabilities. Additionally, the contract maintains a dynamic array variable, IDList, which stores all registered VIDs.

The contract provides an Upload method invoked by an RSU when a vehicle requests to join a VC (see Section 4.4). The RSU uses the Upload method to store the requesting vehicle’s CredID and Source information in the IDList array on the blockchain.

#### 3.2.2. Vehicular Cloud Smart Contract (VCSC)

We design a VCSC to securely manage essential VC-related data. It is deployed for each VC and maintained by the corresponding cloud administrators. The formal definition of the VCSC is provided in Appendix C (see Algorithm 5).

Specifically, the contract declares several VC-related data (variables): VID, representing the unique identifier of the VC; ADM and MEM, arrays holding the identifiers of cloud administrators and members, respectively; MAP, which records the mapping from PBT leaf nodes to vehicle identifiers; and ProMess, a dynamic array storing proposal messages, along with their processing status.

The contract provides the following functions:

- Update: Invoked by cloud administrators to initialize VC-related variables;
- Upload: Invoked by vehicles or administrators to append new proposal messages;
- Return: Invoked by administrators to retrieve unprocessed proposal messages.

## 4. Blockchain-Based Dynamic Key Management (BcDKM) Scheme

### 4.1. High-Level Description

**Figure 2:**
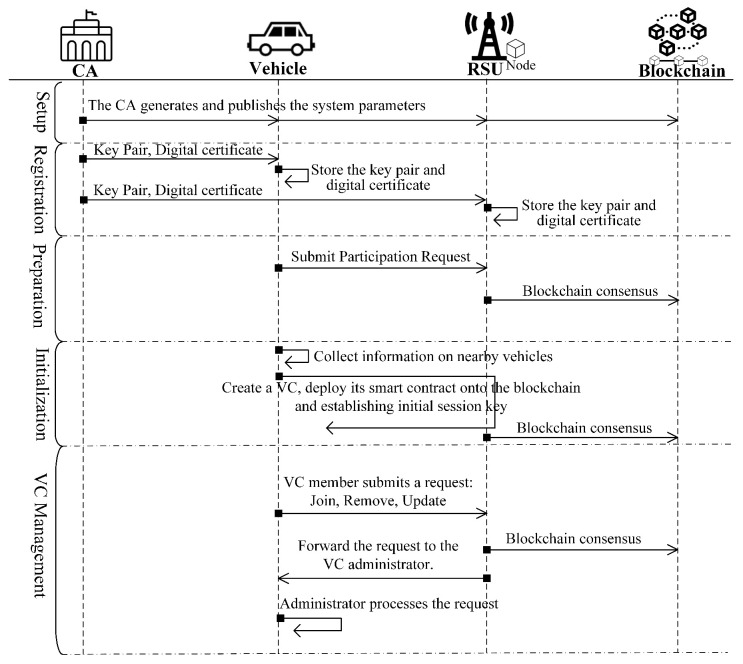
High-level workflow of the BcDKM scheme.

Our proposed blockchain-based dynamic key management (BcDKM) scheme for vehicle cloud (VC) systems consists of several phases: In the **Setup** phase, the Certification Authority (CA) initializes the entire system by establishing essential system parameters. The subsequent **Registration** phase involves vehicles and RSUs generating cryptographic keys and registering with the CA to obtain certificates. During the **Preparation** phase, registered vehicles submit participation requests to local RSUs, which verify and record vehicle information on the blockchain. The **Initialization** phase allows vehicles to initialize VCs by deploying smart contracts and establishing initial session keys. Finally, the **VC Management** phase supports dynamic operations such as allowing external vehicles to securely Join an existing VC, enabling current members to securely Remove themselves from a VC, and facilitating secure and dynamic group key Update operations among existing members. An overview of the BcDKM scheme phases is depicted in Figure 2.

### 4.2. Setup

Upon input of $1^{\kappa }$, the CA performs the following steps:

1. Invoke the **SetUp**(κ) algorithm of the CGKAwAM (see Definition 3) to obtain the common system parameters ($csp=(\mathrm{PKE},H_{PRG},psp)$).
2. Select a digital signature scheme (DS={DInit,DGen,DSign,DVer}). Invoke $\mathrm{DInit}(1^{\kappa })$ to obtain the dsp parameters, and invoke DGen(dsp) to generate a key pair $\left(sk_{ca},pk_{ca}\right)$ (See Definition 2).
3. Select a symmetric encryption scheme (SE={SGen,SEnc,SDec}. Invoke $\mathrm{SGen}(1^{\kappa }$) to generate a secret key (*k*).
4. Initialize a blockchain system, denoted as BC, which supports the VCSC for the management of VC-related information and the VIDSC for the recording of information of vehicles willing to join a VC. Those smart contracts are defined in Section 3.2.
5. Publish the system parameter as sp = (csp, DS, SE,BC, $dsp,ssp,pk_{ca})$. We assume sp is pre-stored in vehicles and RSUs.

### 4.3. Registration

**Figure 3:**
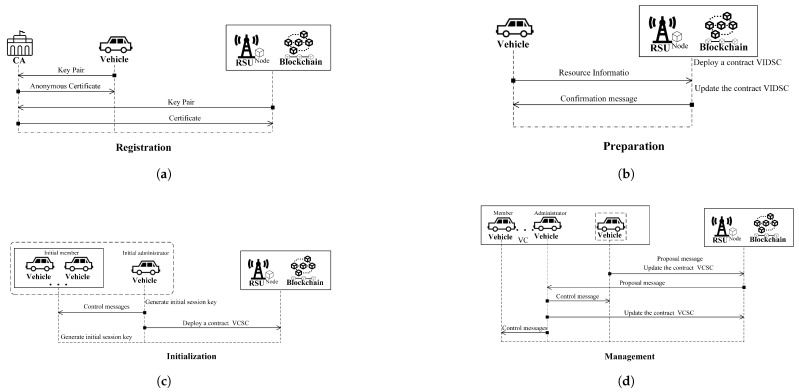
Message sequence of different procedures: (**a**) registration; (**b**) preparation; (**c**) initialization; (**d**) VC management.

Before joining the system, vehicles and RSUs must register with the CA. For a vehicle ($V_{i}$), let $ID_{V_{i}}$ denote its true identity. Each vehicle uses a unique anonymous certificate when participating in different VCs. Typically, a vehicle can obtain multiple such certificates from the CA during a single registration process. As an example, we describe the process in which a vehicle $V_{i}$ registers with the CA and obtains one anonymous certificate. The message transmission sequence of this procedure is illustrated in Figure 3a.

1. $V_{i}$ invokes $\mathrm{KeyGen}(csp,V_{i})$ to generate a private–public key pair, i.e., $KP_{V_{i}}=\left(sk_{V_{i}},pk_{V_{i}}\right)$, and sends $Re_{i}=ID_{V_{i}}\;\|\;pk_{V_{i}}$ to the CA via a secure channel. For simplicity, we assume that the key pair is used for both CGKAwAM and digital signature schemes.
2. Upon receiving $Re_{i}$, the CA does the following:
  (a) Issue an anonymous certificate ($C_{V_{i}}$ = $(pse_{V_{i}}$, $pk_{V_{i}}$, $vp_{V_{i}}$, $sig_{V_{i}})$, where $pse_{V_{i}}$ is a pseudonym generated by the CA by running $\mathrm{SEnc}(k,ID_{V_{i}}∥sn_{i})$, $sn_{i}$ is a serial number, $vp_{V_{i}}$ denotes the validity period, and $sig_{V_{i}}$ is a signature generated by running $\mathrm{DSign}(sk_{ca},pse_{V_{i}}\;\|\;pk_{V_{i}}\;\|\;vp_{V_{i}})$).
  (b) Send $C_{V_{i}}$ to $V_{i}$ over a secure channel.
3. $V_{i}$ can then easily verify the signature ($sig_{V_{i}}\in C_{V_{i}}$) using the DVer algorithm and store $C_{V_{i}}$ locally.

For an RSU ($R_{i}$), privacy is generally not a concern. RSUs generate their certificates in the same way as vehicles; however, the only difference is that the pseudonym in the certificate is set to the true identity rather than an anonymized identifier. Let $ID_{R_{i}}$ denote the true identity of $R_{i}$. $R_{i}$ generates a private–public key pair ($KP_{R_{i}}=\left(sk_{R_{i}},pk_{R_{i}}\right)$). $R_{i}$ submits $ID_{R_{i}}\;\|\;pk_{R_{i}}$ to the CA via a secure channel and obtains a certificate ($C_{R_{i}}=\left(ID_{R_{i}},pk_{R_{i}},vp_{R_{i}},sig_{R_{i}}\right)$) issued by the CA.

### 4.4. Preparation

In this phase, registered vehicles submit participation requests to their local RSUs. The RSUs are responsible for verifying the vehicles’ identities and recording their information for VC admission. As an example, we describe the process in which a vehicle ($V_{i}$) submits its participation request to RSU $R_{i}$. Suppose that RSU $R_{i}$ deploys a contract (${\mathrm{VIDSC}}_{R_{i}}$) on the blockchain. Let $V_{i}$ and $R_{i}$ hold certificates $C_{V_{i}}$ and $C_{R_{i}}$ and key pairs $\left(sk_{V_{i}},pk_{V_{i}}\right)$ and $\left(sk_{R_{i}},pk_{R_{i}}\right)$, respectively. The message transmission sequence of this procedure is illustrated in Figure 3b. They then proceed as follows:

1. When $V_{i}$ enters the area managed by $R_{i}$, it generates a signature ($S_{V_{i}}$) on the message expressed as $m_{i}=(C_{V_{i}}\;∥\;V_{i}\;∥\;R_{i}\;∥\;ts\;∥\;\mathrm{mess})$ using $\mathrm{DSign}(sk_{V_{i}},m_{i})$, where ts is a timestamp and mess is the description of $V_{i}$’s resource information. Then, $V_{i}$ sends $\left(S_{V_{i}},m_{i}\right)$ to $R_{i}$.
2. Upon receiving $\left(S_{V_{i}},m_{i}\right)$, $R_{i}$ does the following:
  (a) Verify the validity of the signature by invoking $\mathrm{DVer}(pk_{V_{i}},m_{i},S_{V_{i}})$. If the signature is invalid, abort; otherwise, invoke the Upload$\left(C_{V_{i}},\mathrm{mess}\right)$ method (see Section 3.2) within ${\mathrm{VIDSC}}_{R_{i}}$ to store the vehicle information.
  (b) Generate a signature ($Rv_{V_{i}}$) on the message ($mv_{i}=(C_{R_{i}}\;∥\;m_{i}\;∥\;sc)$) using $\mathrm{DSign}(sk_{R_{i}}$, $mv_{i})$, where sc is the address of ${\mathrm{VIDSC}}_{R_{i}}$.
  (c) Send $\left(Rv_{V_{i}},mv_{i}\right)$ to $V_{i}$.
3. $V_{i}$ can easily verify the validity of the received signature by invoking $\mathrm{DVer}(pk_{R_{i}}$, $mv_{i}$, $Rv_{V_{i}})$. Moreover, since the blockchain is public, it is straightforward to check the correctness of the contract address.

### 4.5. Initialization

Suppose a vehicle ($V_{1}$) intends to initialize a VC. As an example, assume that it obtains a set of authenticated vehicle identifiers ($G=\{V_{1},\ldots ,V_{n}\},n>1$), representing the initial set of authenticated vehicle identities acquired from the nearest RSU. Furthermore, suppose that each vehicle $V_{i}\in G$ holds a key pair $\left(sk_{V_{i}},\;pk_{V_{i}}\right)$ and an anonymous certificate ($C_{V_{i}}$). The message transmission sequence of this procedure is illustrated in Figure 3c. $V_{1}$ initializes a VC as follows:

1. Invoke the **Create**$\left(csp,G,V_{1}\right)$ algorithm of CGKAwAM (see Section 3) to obtain the initialization local state ($\gamma_{V_{1}}^{vid}=\left(\mathrm{Role},\mathrm{Adm},\mathrm{Mem},\mathrm{Map},\mathrm{len},n,{\left\{A_{j}\right\}}_{j=1}^{Y},rsk,\mathrm{index},{\mathrm{vcsc}}_{vid}\right)$) and a set of control messages (CM).
2. Deploy a VCSC smart contract (see Section 3.2.2) for the VC identified by vid via the RSU on the blockchain as follows:
  (a) Deploy the ${\mathrm{VCSC}}_{vid}$ on the blockchain. If the deployment is successful, the RSU returns the contract address (${\mathrm{sc}}_{vid}$); otherwise, the process is aborted.
  (b) Invoke the Update(vid,Map,Adm,Mem,n) method within the ${\mathrm{VCSC}}_{vid}$ contract to initialize the VC-related data (see Section 3.2.2).
3. Send each control message (cm∈CM) to its corresponding vehicle in $G\setminus \{V_{1}\}$, attaching the same ${\mathrm{sc}}_{vid}$ to all messages.

When a vehicle ($V_{z}\in G\setminus \left\{V_{1}\right\}$) receives the control message (cm) from the cloud administrator ($V_{1}$), the **Process**$\left(gid,V_{z},\gamma_{V_{z}}^{vid},cm\right)$ algorithm of CGKAwAM (see Definition 3) is invoked to obtain the initialization local state ($\gamma_{V_{z}}^{vid}$ = (Role, Adm, Mem, Map, len, *n*, ${\left\{A_{j}\right\}}_{j=1}^{Y}$, rsk, ip, ${\mathrm{sc}}_{vid})$).

### 4.6. VC Management

This subsection describes how cloud members in a created VC are managed. Specifically, a vehicle may Join a VC as a new member, an existing member may Remove itself from a VC, and a member may trigger a group key Update. All three operations follow a unified procedure, where the main difference lies in the command (cmd) type and, in the case of Update, the inclusion of an additional ciphertext (txt). The message transmission sequence of this procedure is illustrated in Figure 3d. The generalized procedure is expressed as follows:

1. A vehicle ($V_{u}$) invokes the **Proposal**$\left(csp,vid,V_{u},V_{a},\mathrm{cmd},\mathrm{txt}\right)$ algorithm of CGKAwAM (see Section 3) to generate a proposal message ($P=(\mathrm{vid}\;\|\;\mathrm{cmd}\;\|\;V_{a}\;\|\;V_{u}\;\|\;\mathrm{txt})$). Here, cmd∈{ADD,REM,UPD} correspond to Join, Remove, and Update, respectively. For Join and Remove, txt=null, while for Update, $\mathrm{txt}=\mathrm{PEnc}(pk_{V_{a}},sd)$, where sd is a random number selected by $V_{u}$.
2. $V_{u}$ uploads message P to the smart contract (${\mathrm{VCSC}}_{vid}$) through the RSU by invoking the Upload(P,f) method, where f=0.
3. Once message P has been successfully stored, the RSU notifies the administrator ($V_{a}$) to handle the message.
4. $V_{a}$ invokes the Return(cmd) method within ${\mathrm{VCSC}}_{vid}$ to retrieve P.
5. $V_{a}$ runs the **Commit**$\left(csp,\gamma_{V_{a}}^{vid},P\right)$ algorithm of CGKAwAM to update its local state ($\gamma_{V_{a}}^{vid}$), yielding a new state (${\gamma^{\prime }}_{V_{a}}^{vid}$) and a set of control messages (CM).
6. $V_{a}$ invokes the Update(vid,Map,Adm,Mem,n) method in ${\mathrm{VCSC}}_{vid}$ to update the VC-related data using ${\gamma^{\prime }}_{V_{a}}^{vid}$.
  - In the case of Join, $V_{u}$ is added to the membership set.
  - In the case of Remove, $V_{u}$ is excluded from the membership set.
7. $V_{a}$ sends each control message (cm∈CM) to the corresponding vehicles. The recipient set differs slightly depending on the operation:
  - Join: all members in $\left\{G\setminus \left\{V_{a}\right\}\right\}\cup \left\{V_{u}\right\}$;
  - Remove: all members in $G\setminus \{V_{a},V_{u}\}$;
  - Update: all members in $G\setminus \{V_{a},V_{u}\}$.
  Each recipient vehicle ($V_{z}$) executes the **Process**$\left(vid,V_{z},\gamma_{V_{z}}^{vid},cm\right)$ algorithm of CGKAwAM to update its local state accordingly.

It is worth noting that in highly dynamic vehicular networks, temporary disconnections of vehicles are inevitable. Nevertheless, the asynchronous design of our scheme ensures that such disconnections do not hinder the execution of group key updates. Specifically, if a vehicle disconnects after submitting a *Proposal* message, the administrator continues processing the request and distributes the corresponding *Control* message to other VC members, who can update the session key locally. The disconnected vehicle may later request the *Control* message upon reconnection or reinitiate its join request. If a vehicle disconnects before its *Proposal* message reaches the VC administrator, no session key update is triggered, and the VC remains unchanged. Therefore, both the correctness and security of the group are preserved, even in the presence of frequent disconnections.

## 5. Security Analysis

This section presents the security analysis of the proposed schemes. First, we formally prove that the CGKAwAM scheme (see Section 3) is secure against adversaries in VC environments and achieves the security properties of known-key security, forward secrecy, and post-compromise security (see Section 2.2), as detailed in Section 5.1. Based on this result, we further demonstrate that the proposed blockchain-based dynamic key management (BcDKM) scheme for VC systems (see Section 4) achieves the intended security goals, as shown in Section 5.2.

### 5.1. Security Analysis of CGKAwAM

#### 5.1.1. Security Definition and Model

**Definition** **6** (**Game-Based Security Notions**)**.** *We capture the security of our CGKAwAM through a security game played between an adversary (*A*) and a challenger (* C*). In the security game,* C *simulates the execution of the scheme, while* A *interacts with the oracles (see Definition 7) maintained by* C*, through issuing queries (see Definition 8).*

**Definition** **7** (Oracles)**.** *We consider μ users (e.g., vehicles), i.e., $V_{1},\ldots ,V_{\mu }$ in this system. Each user ($V_{i}$) is represented by a set of oracles, denoted as $\pi_{V_{i}}^{s}$, which corresponds to the s-th group session that $V_{i}$ participates in. Furthermore, we use the $\pi_{V_{i}}^{s}\left[t\right]$ symbol to denote the t-th stage of oracle (group session) $\pi_{V_{i}}^{s}$, where s,t∈N. A stage corresponds a specific key update within a group session. Each time the group executes a proposal message (e.g., member join, leave, or session key update), a new stage is triggered, and the session key is updated accordingly. For example, $\pi_{V_{i}}^{s}\left[0\right]$ represents the initial establishment of a group session and the computation of the corresponding initial group session key. Each oracle ($\pi_{V_{i}}^{s}$) has access to the key pair of the $V_{i}$: $\left(sk_{V_{i}},pk_{V_{i}}\right)$. The oracle ($\pi_{V_{i}}^{s}$) is associated with a collection of variables (initially set to empty), defined as follows:*

- *$\pi_{V_{i}}^{s}.gid$ represents the identifier of the group session ($\pi_{V_{i}}^{s}$).*
- *$\pi_{V_{i}}^{s}.role\in \left\{A,M\right\}$ represents the role of $V_{i}$ in the group session ($\pi_{V_{i}}^{s}$). If $\pi_{V_{i}}^{s}.role=A$, the user is the administrator; otherwise, the user is the group member.*
- *$\pi_{V_{i}}^{s}.uid$ represents the number of stages that have occurred in the group session ($\pi_{V_{i}}^{s}$) since its creation. This variable is used solely within the security model.*
- *$\pi_{V_{i}}^{s}\left[t\right].pid$ represents a set containing the identifiers of the users in the group session ($\pi_{V_{i}}^{s}$) at stage t with whom $V_{i}$ intends to establish a session key, including $V_{i}$ himself.*
- *$\pi_{V_{i}}^{s}\left[t\right].gsk$ represents the group session key associated with the group session ($\pi_{V_{i}}^{s}$) at stage t.*
- *$\pi_{V_{i}}^{s}\left[t\right].st\in \left\{accepted,unaccepted\right\}$ represents the status associated with group session $\pi_{V_{i}}^{s}$ at stage t. If the status is accepted, the session key ($\pi_{V_{i}}^{s}\left[t\right].gsk$) has been computed; otherwise, the status is unaccepted.*
- *$\pi_{V_{i}}^{s}\left[t\right].sa$ represents the local state associated with group session $\pi_{V_{i}}^{s}$ at stage t. It corresponds to the γ state in our scheme (see Section 3).*
- *$\pi_{V_{i}}^{s}\left[t\right].ms$ represents the messages sent and received in group session $\pi_{V_{i}}^{s}$ at stage t.*

**Definition** **8** (Query)**.** *We formalize the security game using the oracles defined above. During the simulation of the security game, C allows A to issue the following queries:*

- *$Q_{\mathrm{Setup}}\left(\kappa \right)$: This query models the Setup algorithm of CGKAwAM. Upon receiving the security parameter (κ), C simulates the CA to generate the common system parameter (csp).*
- *$Q_{\mathrm{KG}}\left(csp,V_{i}\right)$: This query models the KeyGen algorithm of CGKAwAM. Upon receiving the csp and a user identifier ($V_{i}$) chosen by A, C returns to A the key pair ($pk_{V_{i}},sk_{V_{i}}$) associated with the user identified by $V_{i}$.*
- *$Q_{\mathrm{Create}}\left(csp,V_{a},G\right)$: This query models the Create algorithm of CGKAwAM. In this query, A is allowed to select an initial administrator ($V_{a}$) to cr(eate a VC with a group of users $G=\{V_{1},\ldots ,V_{n}\}$, where $V_{a}\in G$). Then, C initializes the oracle ($\pi_{V_{a}}^{s}$) and outputs a set of control messages.*
- *$Q_{\mathrm{Prop}}\left(csp,gid,V_{i},V_{a},prop,txt\right)$: This query models the Proposal algorithm of CGKAwAM. In this query, A is allowed to submit a proposal message ($P=(gid\;\|\;prop\;\|\;V_{a}\;\|\;V_{i}\;\|\;txt)$), which suggests that the administrator ($V_{a}$) execute a command (prop∈{ADD,REM,UPD}). C then uploads the proposal message to the smart contract.*
- *$Q_{\mathrm{Comm}}\left(csp,\pi_{V_{a}}^{s},P\right)$: This query models the Commit algorithm of CGKAwAM. In this query, A is allowed to request the administrator ($V_{a}$) to execute the proposal message (P) within the session ($\pi_{V_{a}}^{s}$). C updates the variables associated with the oracle ($\pi_{V_{a}}^{s}$) and outputs a set of control messages.*
- *$Q_{\mathrm{Proc}}\left(gid,V_{i},\pi_{V_{i}}^{s},\mathrm{cm}\right)$: This query models the Process algorithm of CGKAwAM. In this query, A is allowed to request the user ($V_{i}$) to execute the control message (cm). C updates the variables associated with the oracle ($\pi_{V_{i}}^{s}$).*
- *$Q_{\mathrm{Rev}}\left(\pi_{V_{i}}^{s},t\right)$: This query models the adversary’s ability to reveal a group session key in session $\pi_{V_{i}}^{s}$ at stage t. C returns the group session key ($\pi_{V_{i}}^{s}\left[t\right].gsk$).*
- *$Q_{\mathrm{Cor}}\left(\pi_{V_{i}}^{s},t\right)$: This query models the adversary’s ability to compromise the user ($V_{i}$) in session $\pi_{V_{i}}^{s}$ at stage t. C returns all the values stored in session $\pi_{V_{i}}^{s}\left[t\right]$ at stage t, except the group session key.*
- *$Q_{\mathrm{Test}}\left(\pi_{V_{i}}^{s},t\right)$: It requires that $\pi_{V_{i}}^{s}\left[t\right].st=accepted$ and the output of $\mathrm{Freshness}(\pi_{V_{i}}^{s},t)$ be fresh (see Definition 10). If either of these two conditions is not satisfied, the query outputs ⊥. Otherwise, the output of this query is determined as follows: If b=0 (where the bit (b) is defined in Definition 9), it outputs $\pi_{{\mathrm{ID}}_{i}}^{s}\left[t\right].gsk$; otherwise (i.e., b=1), it outputs a random session key.*

**Definition** **9** (Security Model)**.** *We define our security model through a security game played between an adversary (A) and a challenger (C). In the security game, C simulates the execution of the scheme using oracles (see Definition 7), while A interacts with the oracles by issuing queries (see Definition 8). We further define a non-adaptive security model to formalize the security of our scheme. In this model, the adversary (A) is considered non-adaptive, meaning that it must determine all of its queries in advance, before the security game begins. Once the queries are fixed, A is no longer allowed to modify any query or its target. The security game is defined as follows:*

*1*. *Before C begins simulating the security game, it chooses a random bit (b∈{0,1}).*
*2*. *A outputs a fixed query set of $Q=\{q_{1},\ldots ,q_{\alpha }\}$ to C, specifying the exact sequence of queries it intends to issue. Each query ($q_{i}\in Q$) corresponds to a specific query type defined in Definition 8. Note that the $Q_{\mathrm{Setup}}$ and $Q_{\mathrm{Test}}$ queries are each restricted to be issued once, at most.*
*3*. *Once the game begins, C allows A to issue only the predefined queries in set Q and returns the corresponding responses.*
*4*. *After completing all queries, A outputs a bit ($b^{\prime }\in \left\{0,1\right\}$). If $b^{\prime }=b$, we say that A wins the game. The advantage of A is defined as ${\mathrm{Adv}}_{A}=\left|\mathrm{Pr}\left[\mathrm{Wins}\right]-\frac{1}{2}\right|$.*

**Definition** **10** (Freshness)**.** *Freshness is used to prevent the adversary from trivially winning the game described in the security model. We define $\mathrm{Freshness}(\pi_{V_{i}}^{s},t)$ as fresh if and only if the following boolean expression holds: $F_{1}\wedge F_{2}\wedge F_{3}\vee F_{4}=1,$ where $F_{1}$, $F_{2}$, $F_{3}$, and $F_{4}$ are boolean predicates defined as follows:*

*1*. *$F_{1}$: If $\pi_{V_{i}}^{s}\left[t\right].st=\mathrm{accepted}$, then set $F_{1}=1$. Otherwise, set $F_{1}=0$.*
*2*. *$F_{2}$: If the adversary has not issued query $Q_{\mathrm{Rev}}\left(\pi_{V_{i}}^{s}t\right)$ and has not issued $Q_{\mathrm{Rev}}$ for any stage that is a partner of $\pi_{V_{i}}^{s}\left[t\right]$ (see Definition 11), then set $F_{2}=1$; otherwise, set $F_{2}=0$. $F_{2}=1$ ensures that the group session key is not trivially accessible by the adversary and is used to capture the notion of known-key security.*
*3*. *$F_{3}$: If the adversary has not issued query $Q_{\mathrm{Crr}}\left(\pi_{V_{i}}^{s},t\right)$ and has not issued $Q_{\mathrm{Crr}}$ for any stage that is a partner of $\pi_{V_{i}}^{s}\left[t\right]$ (see Definition 11), prior to $\pi_{V_{i}}^{s}\left[t\right].st=\mathrm{accepted}$, and the same condition holds for all related stages with t>0, then set $F_{3}=1$; otherwise, set $F_{3}=0$. $F_{3}=1$ is used to capture forward secrecy.*
*4*. *$F_{4}$: For any $V_{j}\in \pi_{V_{i}}^{s}\left[t\right].pid$, if $V_{j}$ has been queried by $Q_{\mathrm{Crr}}$ at any time prior to $\pi_{V_{i}}^{s}\left[t\right]$, then there must exist a stage ($\pi_{V_{j}}^{s^{\prime }}\left[t^{\prime }\right]$) such that*
  - *$\pi_{V_{j}}^{s^{\prime }}\left[t^{\prime }\right].uid<\pi_{V_{i}}^{s}\left[t\right].uid$, indicating that both stages belong to the same group and that $\pi_{V_{j}}^{s^{\prime }}\left[t^{\prime }\right]$ occurred earlier than $\pi_{V_{i}}^{s}\left[t\right]$.*
  - *The $\pi_{V_{j}}^{s^{\prime }}\left[t^{\prime }\right]$ stage corresponds to a group key update for $V_{j}$ initiated via a proposal with a UPD command and successfully executed, without being influenced by the adversary.*
  *If this condition is satisfied for all such $V_{j}$ values, set $F_{4}=1$; otherwise, set $F_{4}=0$. $F_{4}=1$ is used to capture post-compromise security.*

*It should be noted that the vehicle ($V_{j}$) targeted by the query ($Q_{\mathrm{Crr}}\left(\pi_{V_{i}}^{s},t\right)$) must not serve as an administrator during the session (i.e., $\pi_{V_{i}}^{s}\left[t\right].\mathrm{role}\neq A$).*

**Definition** **11** (Partner Stage)**.** *Partner stages refer to a set of stages ($S=\{\pi_{V_{i}}^{s}\left[t\right],\ldots ,\pi_{V_{j}}^{u}\left[v\right]\}$) that correspond to the stages held by all members of the same group at a specific point in time. We define the stages in S to be partner stages of each other if, for any two stages ($\pi_{V_{i}}^{s}\left[t\right],\pi_{V_{j}}^{u}\left[v\right]$), the following conditions hold:*

*1*. *They have the same status, i.e., $\pi_{V_{i}}^{s}\left[t\right].st=\pi_{V_{j}}^{u}\left[v\right].st$.*
*2*. *They are associated with the same group identifier, i.e., $\pi_{V_{i}}^{s}\left[t\right].vid=\pi_{V_{j}}^{u}\left[v\right].vid$.*
*3*. *They share the same participant list, i.e., $\pi_{V_{i}}^{s}\left[t\right].pid=\pi_{V_{j}}^{u}\left[v\right].pid$.*
*4*. *They correspond to the same update stage of the group, i.e., $\pi_{V_{i}}^{s}\left[t\right].uid=\pi_{V_{j}}^{u}\left[v\right].uid$.*

#### 5.1.2. Security Proof

**Theorem** **1.** *Assume that PKE is a public-key encryption scheme that is secure under chosen plaintext attacks (CPAs) and that $H_{PRG}$ is a secure pseudorandom generator. Then, for the adversary (A) defined in our non-adaptive security model, the advantage of A in winning the security game is bounded as follows:*

(1) $${\mathrm{Adv}}_{A}\leq \left(2^{d}-1\right)\cdot \left({\mathrm{Adv}}_{A}^{\mathrm{PRG}}+{\mathrm{Adv}}_{A}^{\mathrm{CPA}}\right)+\frac{1}{2}.$$

*where $d=\lceil \log_{2}\left(n\right)\rceil +1$ and n denotes the maximum number of users allowed in a group.*

For completeness, the full proof of Theorem 1 is presented in Appendix D.

### 5.2. Security Analysis of the Blockchain-Based Dynamic Key Management (BcDKM) Scheme

This section evaluates the security of our proposed blockchain-based dynamic key management (BcDKM) scheme for VC systems (see Section 4). Specifically, our BcDKM scheme is primarily built upon the CGKAwAM scheme. In addition, it incorporates symmetric encryption and blockchain technology to enhance vehicle privacy and improve the manageability of vehicular clouds (VCs). Symmetric encryption is employed to construct anonymous certificates, with the private key generated and securely maintained by a trusted CA for encryption of the real identities of vehicles. Since this key is known only to the CA, the use of symmetric encryption does not introduce additional security risks. Moreover, to support decentralized VC discovery and management, blockchain technology is leveraged to record VC and vehicle-related information. As outlined in the threat model (see Section 2.2), we assume that the blockchain provides basic security guarantees. Under this assumption, even if an adversary compromises some RSUs functioning as blockchain nodes, it remains infeasible to gain control over the entire blockchain and thereby tamper with VC and vehicle data. Therefore, such attacks do not pose a substantive threat to the security of our scheme. We demonstrate that our BcDKM scheme meets the intended design goals and security requirements, as formally proven in the following theorem. For completeness, detailed proofs of all theorems are provided in Appendix D.

**Theorem** **2.** *The proposed BcDKM scheme satisfies vehicle privacy.*

**Theorem** **3.** *The proposed BcDKM scheme satisfies known-key security.*

**Theorem** **4.** *The proposed BcDKM scheme satisfies forward secrecy.*

**Theorem** **5.** *The proposed BcDKM scheme satisfies post-compromise security.*

**Theorem** **6.** *The proposed BcDKM scheme achieves distributed VC discovery.*

**Theorem** **7.** *The proposed BcDKM scheme achieves distributed management.*

**Theorem** **8.** *The proposed BcDKM scheme achieves round optimality.*

**Theorem** **9.** *The proposed BcDKM scheme achieves large-scale VC scalability.*

**Theorem** **10.** *The proposed BcDKM scheme supports asynchrony.*

## 6. Performance Analysis

In this section, we comprehensively evaluate the performance of our proposed BcDKM scheme from three perspectives. First, in Section 6.1 (Functional Comparison), we compare the functional characteristics of our scheme with those of several related key management schemes. Next, in Section 6.2 (Computation and Communication Overhead), we provide a theoretical analysis and comparison of the computational and communication costs between our scheme and the benchmark schemes. Finally, in Section 6.3 (Simulation), we conduct simulations to validate the efficiency and practicality of our scheme.

### 6.1. Functional Comparison

In this section, we compare our scheme with several representative works, focusing on whether they meet the design goals outlined in Section 2.2. Specifically, our primary contribution lies in designing the CGKAwAM group key agreement scheme, and building on this foundation we further develop the BcDKM key management scheme for VC systems. Therefore, when selecting comparison schemes, we primarily consider the representative schemes discussed in Section 1.1 that are either designed for VC systems or serve as potential solutions from different categories of GKM schemes. Specifically, we select AGKA [17], ER-CGKA [22], TGKA [12], and NIGKA [15] as representative GKA-based GKM schemes; GKD [26] as a representative GKD-based GKM scheme; and BcGKM [28] as a recently proposed blockchain-based GKM scheme. AGKA [17] is a bilinear pairing-based group key agreement scheme for vehicular networks, but it requires the group size to be fixed during initialization, which limits scalability. ER-CGKA [22] is built on the CGKA scheme for vehicular scenarios but lacks administrator-driven group management. TGKA [12] is a classical tree-based group key agreement that requires multiple rounds of interaction when group membership changes, resulting in lower communication efficiency. NIGKA [15] is a representative non-interactive group key agreement but only supports static groups with fixed membership. GKD [26] is a key distribution-based scheme for vehicular networks, but it suffers from inherent centralization issues. BcGKM [28] is a recently proposed blockchain-based key management scheme for VCs; however, its key management lacks asynchronous capabilities.

In Table 1, we compare the features and security properties of the selected schemes, focusing on the following aspects: *vehicle privacy, known-key security*, *forward secrecy*, *post-compromise security*, *distributed VC discovery*, *distributed management*, *round optimality*, and *large-scale VC scalability* (as described in Section 2.2). In the Table 1, the “✓” symbol indicates the feature is supported, and “×” indicates it is not supported.

As shown in Table 1, all schemes satisfy the fundamental requirement of *known-key security*. However, TGKA and NIGKA focus solely on cryptographic constructions, without explicit consideration of VC scenarios, and, therefore, do not support *vehicle privacy*. Only ER-CGKA and our scheme achieve *forward secrecy* and *post-compromise security* under active adversaries. In addition, only BcGKA and our scheme leverage blockchain technology, enabling RSUs to act as blockchain nodes for *distributed VC discovery*; however, only our scheme supports *distributed management*. GKD and BcGKA require multiple rounds of online interaction for key establishment and updates, failing to achieve *round optimality*. In contrast, the other schemes require, at most, one round of interaction and inherently support asynchronous operations. Moreover, only ER-CGKA and our scheme exhibit logarithmic computational complexity with respect to the number of VC members, thereby achieving *large-scale VC scalability*. In summary, our proposed scheme is the only one that fulfills all the desired properties.

### 6.2. Computation and Communication Overhead

#### 6.2.1. Computation Overhead

In this section, we compare our proposed BcDKM scheme with several recently introduced schemes designed for VC systems, including GKD [26], AGKA [17], and BcGKA [28], as they share similar design goals and requirements. Table 2 and Table 3 present a comparison of the total computation overhead incurred by all members during the *VC Initialization*, *Join*, *Remove*, and *Update* phases.

To facilitate a clearer comparison of computation overheads, we define the following notations and assumptions. We assume that the VC contains *N* vehicles. We also denote the time costs of various cryptographic operations as follows: $T_{Pm}$ for modular exponentiation, $T_{Am}$ for modular addition, $T_{Mm}$ for modular multiplication, $T_{H}$ for a hash function, and $T_{Pa}$ for a pairing operation. Since both our schemes employ a public key encryption scheme, we instantiate the PKE component using the ElGamal encryption scheme [33]. Accordingly, the computational cost of a single encryption operation is estimated as $2T_{Pm}+T_{Mm}$, while the decryption cost is $T_{Pm}+T_{Mm}$. In general, the cost of a pairing operation ($T_{Pa}$) significantly exceeds that of other cryptographic operations. It is followed by modular exponentiation $T_{Pm}$, while the remaining operations (e.g., modular addition and hash functions) incur relatively lower computation overhead.

As shown in Table 2 and Table 3, in the VC initialization phase, our BcDKM scheme achieves the lowest computation overhead of $O\left(N\right)\cdot \left(3T_{Pm}+2T_{Mm}+2T_{K}\right)$, while GKD, AGKA, and BcGKA incur significantly higher costs. Although the GKD scheme exhibits relatively low computation overhead in the VC management phase, it requires $\left(5T_{Pm}+5T_{H}+7T_{Am}+T_{Mm}\right)+O\left(N\right)\cdot \left(3T_{Mm}+T_{Am}\right)$ for the Join phase and $O\left(N\right)\cdot \left(3T_{Mm}+T_{Am}\right)$ for the Remove and Update phases. However, it lacks essential security guarantees, such as forward secrecy and post-compromise security. In addition, the AGKA scheme consistently incurs high computational costs across all phases due to its reliance on a large number of pairing operations ($T_{Pa}$), which are substantially more expensive than other cryptographic primitives.

#### 6.2.2. Communication Overhead

In this section, we compare the communication overhead of our BcDKM scheme with that of GKD [26], AGKA [17], and BcGKA [28]. Table 4 summarizes the number of communication rounds and total messages exchanged by all members in a VC during the *VC Initialization*, *Join*, *Remove*, and *Update* phases. Here, we define one communication round as a single broadcast message sent by a user to all other members in a VC. As shown in Table 4, our scheme achieves optimal communication efficiency in all phases, requiring only one communication round and O(N) total messages per phase, which is on par with optimal schemes such as AGKA. In contrast, GKD and BcGKA incur higher communication overhead in the *VC Initialization* phase.

### 6.3. Simulation

In this section, we first evaluate the computational overhead of cryptographic operations employed in our scheme, followed by a comparative analysis of the overall runtime efficiency against representative existing schemes. We then assess the gas consumption incurred by the smart contracts used in our scheme to demonstrate its practical feasibility on blockchain platforms. Finally, we simulate the message transmission latency of our scheme under vehicular network scenarios.

#### 6.3.1. Computational Efficiency Evaluation

To evaluate the runtime efficiency of the proposed scheme in a vehicular system, we employed a laptop equipped with an AMD Ryzen 7 4800H 2.90 GHz CPU and 16 GB of memory, running Ubuntu 22.04 OS, as a simulation of the in-vehicle environment. All cryptographic operations were implemented in C/C++ using the Miracl C/C++ library [34]. We adopted the finite-field Diffie–Hellman parameters (ffdhe2048) from RFC 7919 [35] to evaluate the computational costs of $T_{Pm}$, $T_{Am}$, and $T_{Mm}$. For pairing operations ($T_{Pa}$), we used BN curves with 128-bit security. The SHA-256 hash function was used to measure the execution time of hash operations, denoted as $T_{H}$. The average computation times of these basic operations were obtained through multiple experimental runs and are summarized as follows: $T_{Pm}\approx 1.1$ ms, $T_{Am}\approx 0.0005$ ms, $T_{Mm}\approx 0.0052$ ms, $T_{H}\approx 0.01$ ms, and $T_{Pa}\approx 3.5$ ms.

**Figure 4:**
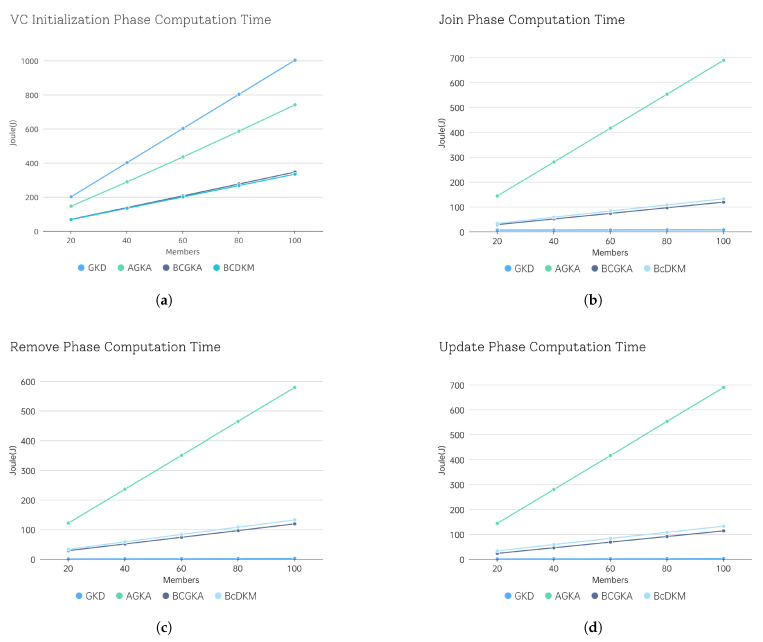
Execution time of different phases: (**a**) VC initialization; (**b**) Join; (**c**) Remove; (**d**) Update.

In Figure 4, we compare the computation time of our proposed BcDKM scheme with that of GKD [26], AGKA [17], and BcGKA [28] across the four respective phases. Specifically, we evaluate the execution time overhead under varying VC member sizes of 20, 40, 60, 80, and 100. As illustrated in the figures, our scheme and BcGKA exhibit lower computational overhead in the VC initialization phase, whereas GKD and AGKA incur significantly higher costs. Although the GKD scheme shows relatively low overhead during the Join, Remove, and Update phases, it lacks essential security properties, as discussed in Section 6.2. In contrast, our scheme is able to complete all four phases, including VC initialization, Join, Remove, and Update, within 1 s, even when the VC size reaches 100 members. These results demonstrate the practical efficiency and effectiveness of our proposed scheme.

**Figure 5:**
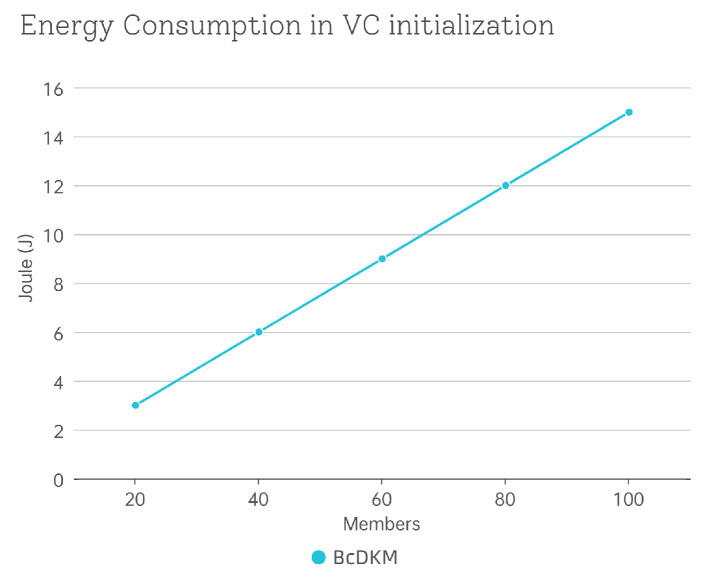
Energy consumption in the VC initialization phase.

To further assess the practicality of our scheme in vehicular systems, we evaluate its energy consumption. The AMD Ryzen 7 4800H 2.90 GHz CPU has a thermal design power (TDP) of 45 W. The energy consumption during the VC initialization phase, which is the most computationally demanding phase of our scheme, is estimated by multiplying the measured execution time by the assumed power. Figure 5 presents the energy consumption for different VC sizes, reported in joules (J).

#### 6.3.2. Smart Contract Overhead Evaluation

We implemented the VIDSC and VCSC smart contracts described in Section 3.2 using Solidity version 0.8.26 and deployed them on a local Ethereum blockchain instance simulated via Remix [36]. The gas consumption for each contract method is summarized in Table 5. The “Deploy” method represents the default cost of deploying each contract. For the VIDSC contract, the deployment consumes 504,155 gas, and the Upload method requires 91,498 gas to store the information of a registered vehicle intending to join a VC during the preparation phase of our scheme. The VIDSC contract does not include the Update or Return method, as these functions are not applicable in this contract. In the case of the VCSC contract, the deployment consumes 1,246,805 gas, under the assumption that each instance manages a VC comprising up to 100 vehicles. The Upload method, used to submit proposal messages, incurs a cost of 185,560 gas per invocation. The Update method, which updates the status of a proposal message, consumes 29,618 gas, while the Return method, which retrieves unprocessed proposal messages matching specific commands, requires 25,193 gas. These two methods are specific to the VCSC contract and are not used in VIDSC.

#### 6.3.3. Network Simulation

To evaluate the message transmission latency of the proposed scheme, we conduct network-level simulations using the NS-3 platform (version 3.43) [37]. The simulations are executed on a laptop equipped with an AMD Ryzen 7 4800H 2.90 GHz CPU and 16 GB of memory, running Ubuntu 22.04 OS. The simulated scenario covers a 1.0×1.0 km^2^ network area, where dynamic nodes (simulating vehicles) exchange messages with static nodes (simulating RSUs) over a wireless channel. The communication range of the nodes is set to 400 m, and the channel bandwidth is limited to 6 Mb/s. Dynamic nodes broadcast messages every 100 ms, with mobility patterns randomly generated at an average speed of 50 km/h.

According to our scheme, the application-layer messages exchanged in the network include Proposal messages and Control messages. The maximum size of a Proposal message is approximately 150 bytes, while that of a Control message is about 200 bytes. The message exchange process required for group establishment and dynamic management is outlined as follows. First, a dynamic node (member) sends a Proposal message to a static node. The static node records the message and notifies the corresponding dynamic node (administrator) to process it and generate the associated Control messages, which are then broadcast to all members. Let $P_{DtS}$ denote the latency of a Proposal message from a dynamic node to a static node. Let $P_{StD}$ denote the latency of a Proposal message from a static node to a dynamic node. Let $C_{DtD}$ denote the latency of a Control message exchanged between dynamic nodes. The experimental results indicate that $P_{DtS}\approx 0.35$ ms, $P_{StD}\approx 0.34$ ms, and $C_{DtD}\approx 0.41$ ms, demonstrating that the message transmission delays in our scheme remain within acceptable limits. These measurements allow us to assess the time required for each phase of the scheme under near-realistic wireless communication conditions.

## 7. Conclusions

In this paper, we proposed a blockchain-based dynamic key management scheme, BcDKM, tailored for VSNs. To support secure and efficient group communication in highly dynamic VC systems, we introduced CGKAwAM, a lightweight extension of the CGKA scheme that enables administrator-driven group management. By integrating CGKAwAM with blockchain technology and smart contracts, the BcDKM scheme achieves decentralized VC discovery/management and asynchronous key updates. We formally proved that the scheme satisfies essential security properties, including key independence, forward secrecy, post-compromise security, and vehicle privacy. Experimental evaluations demonstrated that BcDKM maintains low computational and communication overheads, even for large-scale VCs, and achieves a practical balance between security and performance. These results validate the feasibility and effectiveness of our proposed schemes.

## Figures and Tables

**Table 1:** Comparison of features with those reported in related works.

Property	TGKA [12]	NIGKA [15]	GKD [26]	AGKA [17]	ER-CGKA [22]	BcGKA [28]	Ours
Vehicle privacy	×	×	✓	✓	✓	✓	✓
Known-key security	✓	✓	✓	✓	✓	✓	✓
Forward secrecy	×	×	×	×	✓	×	✓
Post-compromise security	×	×	×	×	✓	×	✓
Distributed VC Discovery	×	×	×	×	×	✓	✓
Distributed Management	×	×	×	×	×	×	✓
Round optimality	✓	✓	×	✓	✓	×	✓
Large-scale VC scalability	×	×	×	×	✓	×	✓

**Table 2:** Computation overheads (I).

Scheme	VC Initialization	Join
GKD [26]	$O\left(N\right)\cdot \left(9T_{Pm}+10T_{H}+14T_{Am}+4T_{Mm}\right)$	$\left(5T_{Pm}+5T_{H}+7T_{Am}+T_{Mm}\right)$ + $O\left(N\right)\cdot \left(3T_{Mm}+T_{Am}\right)$
AGKA [17]	$O\left(N\right)\cdot \left(3T_{Pm}+2T_{Mm}+T_{Pa}\right)$ + $O\left(N^{2}+N\right)\cdot T_{Mm}$ + $2T_{Pa}$	$O\left(N\right)\cdot \left(3T_{Pm}+4T_{Mm}+T_{Pa}\right)+2T_{Pa}$
BcGKA [28]	$O\left(N\right)\cdot (3T_{Pm}+3T_{Mm}+9T_{H}+O\left(N\right)\cdot T_{Am})$	$O\left(N\right)\cdot \left(T_{Pm}+2T_{Mm}+2T_{H}\right)+5T_{Pm}+4T_{H}+2T_{Am}$
Ours	$O\left(N\right)\cdot \left(3T_{Pm}+2T_{Mm}+2T_{H}\right)$	$O\left(N\log_{2}N\right)\cdot T_{H}+O\left(\log_{2}N\right)\cdot \left(2T_{Pm}+T_{Mm}\right)$ + $O\left(N\right)\cdot \left(T_{Pm}+T_{Mm}\right)$

**Table 3:** Computation overheads (II).

Scheme	Remove	Update
GKD [26]	$O\left(N\right)\cdot \left(3T_{Mm}+T_{Am}\right)$	$O\left(N\right)\cdot \left(3T_{Mm}+T_{Am}\right)$
AGKA [17]	$O\left(N\right)\cdot \left(3T_{Mm}+2T_{Pm}+T_{Pa}\right)+2T_{Pa}$	$O\left(N\right)\cdot \left(3T_{Pm}+4T_{Mm}+T_{Pa}\right)+2T_{Pa}$
BcGKA [28]	$O\left(N\right)\cdot \left(T_{Pm}+2T_{Mm}+2T_{H}\right)+5T_{Pm}+4T_{H}+2T_{Am}$	$O\left(N\right)\cdot \left(T_{Pm}+2T_{Mm}+2T_{H}\right)+T_{H}+T_{Am}$
Ours	$O\left(N\log_{2}N\right)\cdot T_{H}+O\left(\log_{2}N\right)\cdot \left(2T_{Pm}+T_{Mm}\right)$ + $O\left(N\right)\cdot \left(T_{Pm}+T_{Mm}\right)$	$O\left(N\log_{2}N\right)\cdot T_{H}+O\left(\log_{2}N+1\right)\cdot \left(2T_{Pm}+T_{Mm}\right)$ + $O\left(N\right)\cdot \left(T_{Pm}+T_{Mm}\right)$

**Table 4:** Communication overhead.

Scheme	VC Initialization		Join		Remove		Update	
Overhead	Rounds	Messages	Rounds	Messages	Rounds	Messages	Rounds	Messages
GKD [26]	4	O(N)·4	1	O(N)	1	O(N)	1	O(N)
AGKA [17]	1	O(N)	1	O(N)	1	O(N)	1	O(N)
BcGKA [28]	2	O(N)·2	1	O(N)	1	O(N)	1	O(N)
Ours	1	O(N)	1	O(N)	1	O(N)	1	O(N)

**Table 5:** Gas Consumption.

Method	Deploy	Upload	Update	Return
**VIDSC**	504,155	91,498	-	-
**VCSC**	1,246,805	185,560	29,618	25,193

## Data Availability

No data were used to support this study.

## Appendix A

**Definition** **A1** (CPA Security)**.** *A PKE scheme is said to be CPA-secure if, for any probabilistic polynomial-time (PPT) adversary (A), the advantage of winning the following game against a challenger (C) is negligible:*

1. *C runs $\mathrm{PInit}(1^{\kappa })\to psp$ and $\mathrm{PGen}\left(1^{\kappa }\right)\to \left(pk,sk\right)$ and sends the psp and public key (pk) to A.*
2. *A outputs two equal-length messages $\left(m_{0},m_{1}\right)$.*
3. *C chooses a random bit (b∈{0,1}), computes the challenge ciphertext ($c^{*}=\mathrm{PEnc}\left(pk,m_{b}\right)$), and sends $c^{*}$ to A.*
4. *A outputs a guess bit ($b^{\prime }$).*

*An adversary (A) wins the game if $b^{\prime }=b$. The advantage of A in winning the game is defined as ${\mathrm{Adv}}_{A}^{\mathrm{CPA}}=\left|\mathrm{Pr}\left[b^{\prime }=b\right]-\frac{1}{2}\right|.$*

**Definition** **A2** (PRG Security)**.** *A PRG is said to be secure if, for any PPT adversary (A), the advantage of winning the following game against a challenger (C) is negligible:*

1. *C chooses a random bit (b∈{0,1}).*
2. *If b=0, C returns $H_{\mathrm{prg}}\left(r\right)$ to A for a uniformly random r∈R; otherwise, it returns a uniformly random value ($r^{\prime }\in K\times R$) to A.*
3. *A outputs a guess bit ($b^{\prime }$).*

*A wins the game if $b^{\prime }=b$. The advantage of A in winning the game is defined as ${\mathrm{Adv}}_{A}^{\mathrm{PRG}}=\left|\mathrm{Pr}\left[b^{\prime }=b\right]-\frac{1}{2}\right|.$*

## Appendix B

**Algorithm A1** ${\mathrm{PBT}}_{\mathrm{Init}}\left({\left\{a_{i}\right\}}_{i=1}^{n}\right)\to {\left\{A_{j}\right\}}_{j=1}^{Y}$
1: **Input**: A set of secret values ${\left\{a_{i}\right\}}_{i=1}^{n}$. 2: **Output**: An array ${\left\{A_{j}\right\}}_{j=1}^{Y}$ representing a PBT. 3: **Set** $h=\lceil \log_{2}\left(n\right)\rceil +1$. 4: **Set** $Y=2^{h}-1$. 5: Allocate an array ${\left\{A_{j}\right\}}_{j=1}^{Y}$ and initialize all $A_{j}\leftarrow ⌀$. ▹*Initialize an empty PBT.* 6: **for** (z=1,…,n) **do** ▹*Initialize leaf nodes of PBT based on secret value ${\left\{a_{i}\right\}}_{i=1}^{n}$.* 7: $s^{\prime }\left|\right|sk\leftarrow H_{PRG}\left(a_{z}\right)$ 8: $pk_{sk}\leftarrow \mathrm{DerivePK}\left(sk\right)$ 9: $A_{z+2^{h-1}-1}\leftarrow \left(sk,pk_{sk},1,1,a_{z}\right)$ 10: **end for** 11: **for** $\left(z=2^{h-1}-1,z\geq 1,z--\right)$ **do** ▹*Initialize the non-leaf node of PBT.* 12: **if** $\left(A_{2z}\neq ⌀\right)$ and $(A_{2z+1}\neq ⌀$) **then** 13: $sv^{\prime }\leftarrow A_{2z+1}$, $sv^{\prime \prime }\left|\right|sk^{\prime \prime }\leftarrow H_{PRG}\left(sv^{\prime }\right)$, $sv^{\prime \prime \prime }\left|\right|sk^{\prime \prime \prime }\leftarrow H_{PRG}\left(sv^{\prime \prime }\right)$ 14: $A_{z}\leftarrow \left(sk^{\prime \prime \prime },pk_{sk^{\prime \prime \prime }},\left\lfloor \log_{2}z\right\rfloor +1,0,sv^{\prime \prime }\right)$ 15: **else if** $\left(A_{2z}\neq ⌀\right)$ and $(A_{2z+1}==⌀$) **then** 16: $sv^{\prime }\leftarrow A_{2z}$, $sv^{\prime \prime }\left|\right|sk^{\prime \prime }\leftarrow H_{PRG}\left(sv^{\prime }\right)$, $sv^{\prime \prime \prime }\left|\right|sk^{\prime \prime \prime }\leftarrow H_{PRG}\left(sv^{\prime \prime }\right)$ 17: $A_{z}\leftarrow \left(sk^{\prime \prime \prime },pk_{sk^{\prime \prime \prime }},\left\lfloor \log_{2}z\right\rfloor +1,0,sv^{\prime \prime }\right)$ 18: **end if** 19: **end for** 20: **return** ${\left\{A_{j}\right\}}_{j=1}^{Y}$.

**Algorithm A2** ${\mathrm{PBT}}_{\mathrm{Ext}}\left({\left\{A_{j}\right\}}_{j=1}^{Y}\right)\to {\left\{A_{k}^{\prime }\right\}}_{k=1}^{2Y+1}$
1: **Input**: An existing PBT ${\left\{A_{j}\right\}}_{j=1}^{Y}$ of height *h*, $Y=2^{h}-1$. 2: **Output**: An extended PBT ${\left\{A_{k}^{\prime }\right\}}_{k=1}^{2Y+1}$ of height h+1. 3: $n\leftarrow 2^{h-1}$ 4: Generate random secret values ${\left\{b_{i}\right\}}_{i=1}^{n}$ 5: ${\left\{B_{j}\right\}}_{j=1}^{Y}\leftarrow {\mathrm{PBT}}_{\mathrm{Init}}\left({\left\{b_{i}\right\}}_{i=1}^{n}\right)$ 6: $sv_{new}∥sk_{new}\leftarrow H_{PRG}\left(B_{1}.sv\right)$ 7: $pk_{sk_{new}}\leftarrow \mathrm{DerivePK}\left(sk_{new}\right)$ 8: $pk_{new}\leftarrow pk_{sk_{new}}$ 9: $A_{1}^{\prime }\leftarrow \left(sk_{new},pk_{new},h+1,0,sv_{new}\right)$ ▹*$A_{1}^{\prime }$ is the new root node.* 10: Attach the tree ${\left\{A_{j}\right\}}_{j=1}^{Y}$ as the left subtree of $A_{1}^{\prime }$ 11: Attach the tree ${\left\{B_{j}\right\}}_{j=1}^{Y}$ as the right subtree of $A_{1}^{\prime }$ 12: The internal structure of each subtree is preserved 13: **return** ${\left\{A_{k}^{\prime }\right\}}_{k=1}^{2Y+1}$

**Algorithm A3** ${\mathrm{PBT}}_{\mathrm{Upd}}\left({\left\{A_{j}\right\}}_{j=1}^{Y},I,ra\right)\to \mathrm{Void}$
1: **Input**: An PBT ${\left\{A_{j}\right\}}_{j=1}^{Y}$, $h=\lceil \log_{2}\left(n\right)\rceil +1$, $Y=2^{h}-1$, and a position *I*. 2: **Output**: ${\left\{A_{j}\right\}}_{j=1}^{Y}$. 3: $sk||sv^{\prime }\leftarrow H_{PRG}\left(ra\right)$ 4: $pk_{sk}\leftarrow \mathrm{DerivePK}\left(sk\right)$ 5: $A_{I+2^{h-1}-1}=\left(sk,pk_{sk},1,1,ra\right)$ 6: **for**$\left(k=I+2^{h-1}-1,k<1,k=k/2\right)$ **do** ▹*Update the nodes along the path from the leaf node $A_{I+2^{h-1}-1}$ to the root node $A_{1}$.* 7: $\left(sk,pk,l,e,sv\right)\leftarrow A_{k}$ 8: $sk^{\prime }\left|\right|sv^{\prime \prime }\leftarrow H_{PRG}\left(sv\right)$, $sk^{\prime \prime }\left|\right|sv^{\prime \prime \prime }\leftarrow H_{PRG}\left(sv^{\prime \prime }\right)$ 9: $A_{k/2}\leftarrow \left(sk^{\prime \prime },pk_{sk^{\prime \prime }},l+1,0,sv^{\prime \prime }\right)$ 10: **end for**

## Appendix C

**Algorithm A4** Vehicle Identity Smart Contract (VIDSC)
1: **Contract** VIDSC { 2: **struct** VID { ▹ *Structure to hold vehicle identity information.* 3: string CredID; ▹ *The (anonymous) certificate of the vehicle.* 4: string Source;} ▹ *The vehicle’s capability information.* 5: VID[] **public** IDList; ▹ *variable: dynamic array of all VID entries.* 6: **function** Upload(string memory credID, string memory source) **public** { 7: VID memory newVID = VID(credID, source); 8: IDList.push(newVID);}

**Algorithm A5** Vehicular Cloud Smart Contract (VCSC)
1: **Contract** VCSC { 2: **struct** PM { ▹ *Structure to represent a proposal message and its status.* 3: string message; 4: uint256 *f*; } 5: PM[] **public** ProMess; ▹ *ProMess is a dynamic array for storing PMs.* 6: uint256 VID, N; 7: string[] **public** ADM, MEM, MAP; 8: **function** Update(Vid, Map, Adm, Mem, n) **public** { 9: VID = Vid; MAP = Map; 10: ADM = Adm; MEM = Mem; N = n; } 11: **function** Upload(string m, uint256 *f*) **public** { 12: Append (m,f) to ProMess; } 13: **function** Return(cmd) **public** { 14: **for each** (m,f)∈ProMess **do** 15: **if** $\left(cmd^{\prime }=cmd\in m\right)$ **and** (f=0) **then** 16: set f=1 and **return** *m*; 17: **Else return** NULL; }}

## Appendix D

**Proof of Theorem 1.** Our security proof follows the game-based technique for group key management in [21], in which security is analyzed by game hopping through a sequence of games. Specifically, we consider a sequence of security games ($\left\{G_{0},G_{1},\ldots ,G_{Final}\right\}$), all defined over the same probability space. The first game ($G_{0}$) corresponds to the original game, which models the interaction between the adversary (A) and the challenger (C) under the non-adaptive security model (see Definition 9). The last game ($G_{Final}$) corresponds to the ideal game, which models A’s interaction with an ideally hybrid game that reveals no secret information. At the beginning of the proof, the challenger (C) starts from $G_{0}$ and hops to a sequence of hybrid games, each obtained by making a slight modification to the previous one. This process continues until we reach the final game ($G_{Final}$), in which the adversary (A) cannot succeed with a probability non-negligibly greater than 1/2. For each hop, the view of A remains computationally indistinguishable between adjacent games. In other words, each hop introduces only a minimal change, and the two neighboring games are computationally indistinguishable under the CPA and PRG security assumption (see Definitions A1 and A2). Game 0. Let Game 0 to be the security game played between A and C as described above (see Definition 9). This game is described as follows:

1. Before C begins simulating the security game, it chooses a random bit (b∈{0,1}).
2. Let $Q=\{q_{1},\ldots ,q_{p}\}$ be the sequence of queries issued by the (non-adaptive) adversary (A).
3. Once the security game begins, C allows A to issue only the predefined queries in the query set (Q) and returns the corresponding responses (see Definition 8). Since we assume A is a non-adaptive adversary, the target group chosen by A is fixed in advance. Considering the targeted group associated with the query set (Q), we describe the execution of the query set issued by A by C as follows:
  - Initialization: The first query ($q_{1}\in Q$) must be the $Q_{\mathrm{Setup}}$ query, which initializes the system parameters and may be issued, at most, once. Subsequently, a subset of queries in Q, including $Q_{\mathrm{KG}}$, is executed to initialize the users. After the system parameters are generated and the users are initialized, the $Q_{\mathrm{Create}}$ query is executed to initialize the target group for A, and this query may also be issued, at most, once.
  - Execution: The following queries in Q are then executed, allowing A to interact with the targeted group. These queries include $Q_{\mathrm{Prop}}$, $Q_{\mathrm{Comm}}$, $Q_{\mathrm{Proc}}$, $Q_{\mathrm{Rev}}$, and $Q_{\mathrm{Cor}}$.
  - Challenge: Finally, the last query ($q_{p}\in Q$) must be the $Q_{\mathrm{Test}}\left(\pi_{V_{i}}^{s},t\right)$ query. This query can be issued, at most, once and must not output ⊥.
4. After completing all queries, A outputs a bit ($b^{\prime }\in \left\{0,1\right\}$).

Let ${\left\{A_{z}\right\}}_{z=1}^{Y}$ denote the PBT consisting of all nodes generated by the latest session key computation completed by user $V_{i}$ up to and including the target session ($\pi_{V_{i}}^{s}$) at stage *t*, and let *d* denote the height of the PBT. Let $Adv_{j}$ be the advantage of A is winning $\left(i.e.,b^{\prime }=b\right)$ in Game *j*. It should be clear that

(A1) $${Adv}_{A}=Adv_{0}+\frac{1}{2}.$$

Game *j*(1≤j≤Final). This game is identical to Game j−1, except that we perform a series of modifications to the PBT (${\left\{A_{i}\right\}}_{i=1}^{Y}$), which has a height of *d*, associated with the target session ($\pi_{V_{i}}^{s}$) at stage *t*. In each game, we replace the outputs of the PRG in one layer of nodes in the PBT with random values, and we also replace the ciphertexts, which are originally generated using the public keys stored at those nodes, with fake, random ciphertexts. These replacements are performed in a bottom-up manner, starting from the leaf nodes and proceeding upward, layer by layer. Specifically, in each hybrid game (*j* (1≤j≤Final)), we target exactly one layer of the PBT for the replacements described above. Since the leaf layer is defined as level 1 (see Definition 5), Game j=1 performs replacements at the leaf nodes, while Game j=d performs replacements at the root node, which resides at level *d*. Thus, Final=d. After applying this series of replacements, the final hybrid game (Final=d) no longer contains any secret values related to the session ($\pi_{V_{i}}^{s}$) at stage *t*, resulting in no advantage for A, i.e., ${\mathrm{Adv}}_{Final}=0$. Throughout this process, the difference between hybrid Game *j* and Game j−1 is limited to the replacement of either PRG outputs or ciphertexts in a single layer of the PBT. Under cryptographic CPA and PRG security assumptions (see Definitions A1 and A2), such replacements should be computationally indistinguishable to any adversary A. To formalize this, we construct a reduction from the ability of A to distinguish between two adjacent hybrid games to breaking the cryptographic CPA and PRG security assumptions. Specifically, if A can distinguish between Game *j* and Game j−1, then we can construct an algorithm that invokes A as a subroutine to break either the PRG security assumption or the CPA security assumption. In particular, if the change between the games involves replacing PRG outputs, we construct an algorithm ($B_{\mathrm{PRG}}$) that invokes A as a subroutine to break the PRG security assumption; if the change involves replacing ciphertexts generated under the public keys stored in the PBT, we construct an algorithm ($B_{\mathrm{CPA}}$) that invokes A as a subroutine to break the CPA security assumption of the PKE scheme. We describe the construction of $B_{\mathrm{PRG}}$ and $B_{\mathrm{CPA}}$ as follows.

- The $B_{\mathrm{PRG}}$ algorithm simulates Game *j* through the following procedure:
  1. Query the challenger in the PRG security game defined in Definition A1 to obtain a sequence of random values $\left(r_{1},r_{2},\ldots ,r_{2^{d-j}}\right)$.
  2. Let $\left\{A_{k}|k\in \left[2^{d-j},\;2^{d-j+1}-1\right]\right\}$ denote the (j)-th level of the PBT (${\left\{A_{z}\right\}}_{z=1}^{Y}$). In this game, any query that uses the PRG to compute on the secret value (sv) in the tuple (sk,pk,h,e,sv) stored in node $A_{k}$ will have its PRG output replaced with the corresponding random value ($r_{k-2^{d-j}+1}$).
  3. For the remainder of the execution, the simulation proceeds identically to Game j−1 and outputs the bit returned by A.
  We now formally analyze the simulation described above and show that it is perfect. Specifically, we prove that the views of A in hybrid Game j−1 and Game *j* are computationally indistinguishable. Observe that the only way for A to distinguish between the two hybrid games is by distinguishing a sequence of random values ($\left(r_{1},r_{2},\ldots ,r_{2^{d-j}}\right)$) from those generated by the PRG $H_{\mathrm{prg}}$. Since the queries issued by A must satisfy the freshness (see Definition 10), it follows from conditions $F_{3}$ and $F_{4}$ with respect to freshness that A is not allowed to obtain any secret values held by users of the group associated with the target session ($\pi_{V_{i}}^{s}$) at stage *t*. In particular, this implies that the secret values stored in node $A_{k}$ of the PBT at level *j* remain unknown to A. We conclude that the above simulation is correct, without $B_{\mathrm{PRG}}$ accessing the seed of the PRG security game.
  Hence, if A can successfully distinguish between Game j−1 and Game *j*, then $B_{\mathrm{PRG}}$ can leverage the advantage of A to break the security of the PRG across multiple instances of the PRG security game, reaching a contradiction. We therefore derive the following advantage bound:
  (A2) $${Adv}_{j-1}\leq {Adv}_{j}+2^{d-j}\cdot {\mathrm{Adv}}_{A}^{\mathrm{PRG}}.$$
- The $B_{\mathrm{CPA}}$ algorithm simulates Game *j* through the following procedure:
  1. Query the challenger in the CPA security game defined in Definition A1 to obtain a sequence of public keys $\left(pk_{1},pk_{2},\ldots ,pk_{2^{d-j}}\right)$.
  2. Let $\left\{A_{k}|k\in \left[2^{d-j},\;2^{d-j+1}-1\right]\right\}$ denote the (j)-th level of the PBT (${\left\{A_{z}\right\}}_{z=1}^{Y}$). In this game, if a query (e.g., $Q_{\mathrm{Comm}}$) involves encrypting a secret value (sv) using the public key stored in node $A_{k}$, the original public key in the tuple (sk,pk,h,e,sv) is replaced with the corresponding public key ($pk_{k-2^{d-j}+1}$).
  3. Set $M_{0}\leftarrow 0$ and $M_{1}\leftarrow sv$.
  4. Send $M_{0},M_{1}$ to the challenger in the CPA security game and receives the ciphertext (c∗).
  5. For the remainder of the execution, the simulation proceeds identically to Game j−1 and outputs the bit returned by A.
  The only difference between Game j−1 and Game *j* is that in Game *j*, for queries (e.g., $Q_{\mathrm{Comm}}$), the resulting ciphertexts are replaced with fake ciphertexts, whereas in Game j−1, they are generated using the public key encryption scheme.
  We now formally analyze the simulation described above and show that it is perfect. Specifically, we prove that the views of A in hybrid Game j−1 and Game *j* are computationally indistinguishable. First, the queries issued by A must satisfy the freshness (see Definition 10), and it follows from conditions $F_{3}$ and $F_{4}$ with respect to freshness that A is not allowed to obtain any secret values held by users of the group associated with the target session ($\pi_{V_{i}}^{s}$) at stage *t*. Since the adversary does not possess the corresponding secret keys, A cannot distinguish between the public keys used in Game j−1 and those replaced in Game *j* with the challenge public key provided by the CPA security game. Observe that the only way for A to distinguish between the two hybrid games is by distinguishing a sequence of ciphertext (c∗) from those generated by the PKE scheme.
  We conclude that the above simulation is correct.
  Hence, if A can successfully distinguish between Game j−1 and Game *j*, then $B_{\mathrm{CPA}}$ can leverage the advantage of A to break the security of the CPA across multiple instances of the CPA security game, reaching a contradiction. Consequently, we derive the following advantage bound:
  (A3) $${Adv}_{j-1}\leq {Adv}_{j}+2^{d-j}\cdot {\mathrm{Adv}}_{A}^{\mathrm{CPA}}.$$

In the Game Final, all PRG outputs in the PBT are replaced with uniformly random values, and all ciphertexts that were originally generated using the public keys stored in the PBT are replaced with encryptions of zero messages. These replacements eliminate any dependence on secret values throughout the tree. As a result, the view of the adversary (A) is completely independent of the underlying secrets. Therefore, A cannot achieve any non-negligible advantage, which implies that

(A4) $${\mathrm{Adv}}_{\mathrm{Final}}=0.$$

Recall that for each hybrid game (Gamej), where 1≤j≤Final=d, we have the following advantage bound:

(A5) $${\mathrm{Adv}}_{j-1}\leq {\mathrm{Adv}}_{j}+2^{d-j}\cdot \left({\mathrm{Adv}}_{A}^{\mathrm{PRG}}+{\mathrm{Adv}}_{A}^{\mathrm{CPA}}\right).$$

For every *j*, 0≤j≤d, we obtain the following:

(A6) $${\mathrm{Adv}}_{0}\leq \sum_{j=1}^{d}2^{d-j}\cdot \left({\mathrm{Adv}}_{A}^{\mathrm{PRG}}+{\mathrm{Adv}}_{A}^{\mathrm{CPA}}\right).$$

Combined with the definition of ${\mathrm{Adv}}_{A}={\mathrm{Adv}}_{0}+\frac{1}{2}$, we derive the following:

(A7) $${\mathrm{Adv}}_{A}\leq \left(\sum_{j=1}^{d}2^{d-j}\right)\cdot \left({\mathrm{Adv}}_{A}^{\mathrm{PRG}}+{\mathrm{Adv}}_{A}^{\mathrm{CPA}}\right)+\frac{1}{2}.$$

Since $\sum_{j=1}^{d}2^{d-j}=2^{d}-1$, we conclude the following:

(A8) $${\mathrm{Adv}}_{A}\leq \left(2^{d}-1\right)\cdot \left({\mathrm{Adv}}_{A}^{\mathrm{PRG}}+{\mathrm{Adv}}_{A}^{\mathrm{CPA}}\right)+\frac{1}{2}.$$

□

**Proof of Theorem 2.** In the Registration phase of our BcDKM scheme, each vehicle anonymously registers with the CA and obtains a pseudonym generated by the CA, which is computed by encrypting the vehicle’s real identity using the CA’s private key. Vehicles use these pseudonyms to participate in the VC system, ensuring that only the trusted CA can link a pseudonym to a vehicle’s real identity. As a result, vehicle privacy is preserved throughout the system. □

**Proof of Theorem 3.** In our BcDKM scheme, the shared group session key negotiated among vehicles acting as cloud members and the cloud administrator within the VC is generated by our CGKAwAM scheme. According to Theorem 1, any adversary characterized by the threat model described in Section 2.2 cannot compromise the security of other independent session keys, even if it obtains a session key of a specific cloud member within a particular VC. This implies that our BcDKM scheme achieves known-key security. □

**Proof of Theorem 4.** Forward secrecy requires that even if a vehicle is compromised by an attacker at a certain point in time, all session keys established prior to the compromise remain confidential. In our BcDKM scheme, the shared group session keys negotiated among vehicles and the cloud administrator within the VC are derived through our CGKAwAM scheme. Since the CGKAwAM scheme has been proven to provide forward secrecy (see Theorem 1), it follows that all previously generated session keys remain secure, even if a vehicle is later compromised. Therefore, the proposed BcDKM scheme satisfies forward secrecy. □

**Proof of Theorem 5.** Post-compromise security requires that if a vehicle is compromised at a certain point in time and the compromised vehicle subsequently performs a session key update that is not influenced by the attacker, then the updated session key remains confidential. In our BcDKM scheme, all session key updates are executed by the CGKAwAM scheme. As proven in Theorem 1, CGKAwAM ensures that newly updated session keys are secure, even if previous session states or keys have been compromised, provided the attacker does not control the update process. Therefore, BcDKM inherits this property from CGKAwAM and satisfies post-compromise security. □

**Proof of Theorem 6.** In our scheme, each VC is associated with a dedicated VCSC smart contract, which is deployed and maintained by the cloud administrators on a distributed blockchain shared among RSUs. These contracts store essential VC metadata, including the VC identifier, administrator and member lists, and mapping information, ensuring that this data is consistently replicated and accessible across all RSUs. Consequently, vehicles entering a new area can directly obtain accurate and up-to-date VC information from the local RSU without relying on any centralized discovery platform. This distributed design removes single points of failure and significantly improves the reliability and efficiency of VC information dissemination and discovery. □

**Proof of Theorem 7.** In our BcDKM scheme, the cloud administrator list (i.e., Adm) is shared among all cloud members, allowing any cloud member within a VC to submit a proposal message to any cloud administrator in the list. The administrator receiving the proposal message then executes the Commit algorithm to process it. This design introduces redundancy and eliminates the reliance on a single cloud administrator. Our BcDKM scheme preserves this feature by recording the Adm list in the VCSC smart contract of each VC. Consequently, proposal messages can be processed by any listed administrator, ensuring system continuity and resilience against individual failures. □

**Proof of Theorem 8.** In our CGKAwAM scheme, all group-related proposals, including Join, Remove, and Update, are initiated by a single user through a proposal message. An administrator then processes the proposal by executing the Commit algorithm and broadcasts the resulting control messages to all relevant group members. Since this proposal-then-commit paradigm completes within a single round of message dissemination, our BcDKM scheme builds upon CGKAwAM and also achieves round optimality for both key establishment and key updating. This one-round design is particularly well-suited for VC environments that demand high responsiveness and minimal coordination overhead. □

**Proof of Theorem 9.** In the CGKAwAM scheme, group key management is organized using a binary tree structure (i.e., PBT), where each user corresponds to a leaf node. This design allows for efficient operations such as key establishment and key update, with both communication and computation overhead growing logarithmically with the number of group members. Since our BcDKM scheme builds upon CGKAwAM, it inherits this scalability property, ensuring that key management remains efficient, even in large-scale VC environments. □

**Proof of Theorem 10.** The CGKAwAM scheme inherently supports asynchrony, meaning that the key update process does not rely on strict message ordering or simultaneous delivery. Any user can asynchronously submit proposal messages (e.g., Join, Remove, or Update) to the group administrator. Building on this feature, our BcDKM scheme inherits the asynchronous capability of CGKAwAM, enabling vehicles to complete state updates and session key updates without requiring real-time online presence or strict synchronization. □
